# Hematological toxicity of [^225^Ac]Ac-PSMA-617 and [^177^Lu]Lu-PSMA-617 in RM1-PGLS syngeneic mouse model

**DOI:** 10.1186/s41181-025-00333-y

**Published:** 2025-03-24

**Authors:** Meryl Maria Vilangattil, Abir Swaidan, Jonathan Godinez, Marco F. Taddio, Johannes Czernin, Christine E. Mona, Giuseppe Carlucci

**Affiliations:** 1 Ahmanson Translational Theranostics Division, Department of Molecular and Medical Pharmacology, David Geffen School of Medicine, UCLA, 650 Charles E Young Dr S, Los Angeles, Los Angeles, California, California 90095 USA; 2 Jonsson Comprehensive Cancer Center, UCLA, Los Angeles, CA USA

**Keywords:** PC, RLT, PSMA-617, Actinium-225, Luetitium-177, Hematotoxicity

## Abstract

**Background:**

Prostate cancer (PC) has a 34% 5-year survival rate after progressing to metastatic castration-resistant prostate cancer (mCRPC), which occurs in 20–30% of cases. Treatments like chemotherapy, immunotherapy, and PSMA-targeted radioligand therapy (RLT) show promise, but challenges remain with tumor resistance, side effects, and dose-limiting toxicity in kidneys and bone marrow. This study investigated the hematotoxicity, treatment efficacy, and recovery after [^177^Lu]Lu-PSMA-617 and [^225^Ac]Ac-PSMA-617 treatment in a syngeneic PC mouse model.

**Method:**

Twenty-five male C57BL/6 mice were inoculated with RM1-PGLS cells and monitored using [^68^Ga]Ga-PSMA-11 PET/CT. The mice were divided into five groups as follows: (1) [^225^Ac]Ac-PSMA-617 treatment with tumors, (2) [^177^Lu]Lu-PSMA-617 treatment with tumors, (3) control group with tumors, (4) [^225^Ac]Ac-PSMA-617 treatment without tumors, and (5) [^177^Lu]Lu-PSMA-617 treatment without tumors. Tumor volume was measured weekly, and animals were sacrificed when tumors reached 1.5 cm³. Endpoint criteria included tumor size, survival, and body mass. Blood samples were collected at different time points to assess blood cell counts and liver and kidney function.

**Results:**

Both treatments significantly slowed tumor progression and extended survival. [^225^Ac]Ac-PSMA-617-treated mice had a median survival of 70 days, compared to 58 days for [^177^Lu]Lu-PSMA-617-treated mice and 30 days for the control group. Tumor volumes were significantly reduced in both treatment groups (*P* < 0.05). Hematological analysis showed that both treatments reduced WBCs, RBCs, and platelets, but values normalized within 35–42 days. Liver and kidney functions remained unaffected, and no significant renal or hepatic toxicity was observed.

**Conclusion:**

Both [^225^Ac]Ac-PSMA-617 and [^177^Lu]Lu-PSMA-617 caused transient hematotoxicity without prolonged effects. The data do not explicitly support the necessity of immunocompetent models for studying therapeutic outcomes in this context. Future studies incorporating immune profiling are warranted to investigate immune system interactions in radioligand therapy further.

**Supplementary Information:**

The online version contains supplementary material available at 10.1186/s41181-025-00333-y.

## Introduction

The most frequent tumor in a man’s urogenital system is prostate cancer (PC), which is also the second most prevalent male malignant tumor overall. In the PC patients population, men will develop metastatic castration-resistant prostate cancer (mCRPC) in 20–30% of cases (Sung et al. 2021). mCRPC is often challenging to control, and life expectancy fails beyond three years (Beer et al. 2017). Cytotoxic chemotherapy, immunotherapy, and androgen deprivation therapy (ADT) are frequent forms of treatment for mCRPC. Still, their effectiveness will be considerably weakened due to tumor resistance (Crawford et al. 2015).

Prostate-specific membrane antigen (PSMA) is a transmembrane protein overexpressed in PC and is a valuable target for both detection and treatment (Tateishi 2020). PSMA is also moderately expressed in other organs such as the kidney, salivary glands, and proximal small intestine (Ghosh and Heston 2004).

PSMA-PET is currently used clinically to assess the presence of metastatic PSMA-positive PC lesions (Calais et al. 2021; Djaileb et al. 2023; Weiner et al. 2024). PSMA expression increases with the severity of the disease (e.g., tumor grade, metastatic disease, reoccurrence, and androgen independence (Ghosh and Heston 2004). Treatment with PSMA radioligand therapy (RLT), such as [^177^Lu]Lu-PSMA-617 or [^225^Ac]Ac-PSMA-617, has shown survival benefits in patients with mCRPC [9–10], higher therapeutic efficacy compared to other treatment approaches and significant quality of life improvements (Calais et al. 2021; Eyben et al. 2020; Alam et al. 2022; Arbuznikova et al. 2023a, b; Fallah et al. 2023).

RLT aims to administer high doses to cancer cells while minimizing radiation to normal tissues. When treated with radioligand therapy, the kidneys and bone marrow are two of the most significant dose-limiting tissues (Groener et al. 2021; Fallah et al. 2023; Hartrampf et al. 2022). However, in the case of [^177^Lu]Lu-PSMA-617, more adverse events related to hematology than impaired renal function have been reported. For instance, in a multicenter trial, Rahbar et al. reported grade 3–4 hematotoxicity in 12% of patients (3% leukopenia, 10% anemia, and 4% thrombocytopenia)(Rahbar et al. 2017). Hematotoxicity can occur for a variety of reasons, including patients with increased metastatic bone involvement, those who received previous taxane chemotherapy, or those who present initial grade 2 cytopenia (Groener et al. 2021). In a pilot prospective clinical trial in chemotherapy-naïve patients with advanced mCRPC, [^225^Ac]Ac-PSMA-617 showed robust therapeutic efficacy against mCRPC (Sathekge et al. 2019). In addition, in relapsing patients following [^177^Lu]Lu-PSMA-617, [^225^Ac]Ac-PSMA-617 therapy showed enhanced PSA response and prolonged survival (Kratochwil et al. 2017). α-therapy can also significantly impact the treatment of micrometastatic cancers that could benefit from the shorter range and intensity of linear energy transfer (LET) to boost effectiveness (Khreish et al. 2020).

To decipher the hematological toxicity associated with [^177^Lu]Lu-PSMA-617 and [^225^Ac]Ac-PSMA-617, we evaluated a syngeneic mouse model of PC. The overall effectiveness [^177^Lu]Lu-PSMA-617 and [^225^Ac]Ac-PSMA-617 was assessed by monitoring changes in blood parameters like white blood cells (WBC), lymphocytes (LYM), monocytes (MON), neutrophils (NEU), red blood cells (RBC), hemoglobin (HGB), platelets (PLT), mean platelet volume (MPV) and platelet distribution width (PDW) along with several other metrics, including kidney function test parameters like creatinine (CRE) and blood urea nitrogen (BUN) and liver function test parameters like alanine transaminase (ALT), aspartate transaminase (AST), alkaline phosphatase (ALP), total bilirubin (TBIL), albumin (ALB) and total protein (TP).

## Materials and methods

### Radiochemistry

For [^177^Lu]Lu-PSMA-617, the non-carrier-added [^177^Lu]LuCl_3_ was obtained from Eczacbaş Monrol in Turkey, while the PSMA-617 precursor was purchased from WuXi AppTec in China. Before usage, the precursor was stored in aliquots of 0.1% aqueous trifluoroacetic acid (1 mg/mL). For radiolabeling, 5 µg of the precursor was combined with 400 MBq of [^177^Lu]LuCl_3_ (molar activity: 1.05 × 10^18^ Bq/mol) and 0.4 M sodium acetate buffer, pH 4.8, containing dihydroxybenzoic acid (10 mg/mL). The mixture was heated to 95 °C for 15 min, after which it was allowed to cool, and saline was added to dilute it. Without additional purification, the radiochemical purity was ≥ 99%. For [^225^Ac]Ac-PSMA-617, WuXi AppTec (Shangai, China) provided the PSMA-617 precursor, and Oak Ridge National Laboratory (TN, USA) supplied the [^225^Ac]Ac(NO_3_)_3_. [^225^Ac]Ac(NO_3_)_3_ was dissolved in 0.1 M HCL (1 mCi/mL) before its use. Before usage, the precursor was kept in aliquots of 0.1% aqueous trifluoroacetic acid (1 mg/mL). For radiolabeling, 7 µg of precursor was added to 400 kBq of [^225^Ac]AcCl_3_ (molar activity: 5.71 × 10^13^ Bq/mol) and was mixed with 1 M sodium acetate buffer containing dihydroxybenzoic acid (10 mg/mL). The mixture was heated for 30 min to 95 °C (pH ~ 5.4), then cooled and formulated with saline. Without subsequent purification, the radiochemical purity was ≥ 98%.

## In vivo studies

All animal studies were approved by the UCLA Animal Research Committee (approval 2005-090). Mice were housed under pathogen-free conditions with food and water ad libitum and a 12 h–12 h light-dark cycle. Veterinary staff and investigators observed the mice daily to ensure animal welfare. RM1-PGLS cells were obtained from the Memorial Sloan Kettering Cancer Center. The RM1-PGLS cell line is a syngeneic prostate cancer model derived from the RM1 cell line, originally established from a spontaneous prostate tumor in C57BL/6 mice. The “PGLS” modification in the RM1-PGLS line enhances the expression of prostate-specific membrane antigen (PSMA), making it an ideal model for evaluating PSMA-targeted radioligand therapies. The cell line was transfected to overexpress PSMA, which was confirmed by flow cytometry. RM1-PGLS cells were cultured in RPMI-1640 medium supplemented with 10% fetal bovine serum, 1% penicillin-streptomycin, and 2 mM L-glutamine under standard conditions. Inoculation was done subcutaneously (0.1 × 10^6^ cells) into the shoulder region of 6–8 weeks old C57BL/6 mice seven days before starting RLT. PET/CTs were conducted one day before the therapy with [^68^Ga]Ga-PSMA-11. Two groups of mice were treated with 30MBq of [^177^Lu]Lu-PSMA-617 and another two groups with 30 kBq of [^225^Ac]Ac-PSMA-617 along with a control group **(**Table 1**)**. To ensure consistency in precursor dosage, each mouse received 2.86 nmol of ^177^Lu-PSMA (30 MBq) and 0.526 nmol of ^225^Ac-PSMA (30 kBq), based on their respective molar activities of 1.05 × 10^18^ Bq/mol and 5.71 × 10^13^ Bq/mol. Each subset was constituted of 5 mice for a total of *n* = 25 for the study. The tumor volume (TV = 0.5 × (LW2) was measured for groups with tumors every week from inoculation until the end of the study. Tumor growth was measured weekly due to the relatively slow progression in this model, particularly at the lower doses used. This frequency allowed for consistent monitoring while minimizing animal distress and is a common practice in preclinical studies to avoid high variability. The endpoint criteria included tumor volume, survival, and body weight. Animals were sacrificed when the tumor volume reached 1.5 cm^3^ for the tumor groups, followed by the non-tumor groups when the endpoint was reached.

**Table 1:** Study design overview detailing the experimental groups used in the study

Group	Tumor Presence	Treatment	Total Mice
Group 1	With Tumor	[^225^Ac]Ac-PSMA-617	5
Group 2	With Tumor	[^177^Lu]Lu-PSMA-617	5
Group 3	With Tumor	Control (no treatment)	5
Group 4	No Tumor	[^225^Ac]Ac-PSMA-617	5
Group 5	No Tumor	[^177^Lu]Lu-PSMA-617	5

Group 1 to Group 3 consists of tumor-bearing mice treated with either [225Ac]Ac-PSMA-617, [177Lu]Lu-PSMA-617, or no treatment (control), respectively. Groups 4 and 5 include tumor-free mice treated with [225Ac]Ac-PSMA-617 or [177Lu]Lu-PSMA-617. Each group of mice was monitored for tumor growth, hematological parameters, and organ function, with tumor progression, blood cell counts, and kidney and liver function assessed at various time points

## Blood sampling and counting

Blood samples were collected at different time points (days 4, 10, 15, 21, 28 until the endpoint was reached). Samples were collected retro-orbitally in a potassium EDTA-coated tube for counting. To determine hematology parameters and potential toxicity arising from treatment, 50 µl (2 × 25 µl) of whole blood was analyzed using the Abaxis VetScan HM5 hematology analyzer (Allied Analytic, Tampa, FL, USA). Each sample was analyzed to produce a complete, 24-parameter, five-part differential blood count (CBC), including the following measured or calculated parameters: total white blood cell count, lymphocyte count, monocyte count, neutrophil count, eosinophil count, basophil count, lymphocyte percentage, monocyte percentage, neutrophil percentage, eosinophil percentage, basophil percentage, red blood cell count, hemoglobin, hematocrit, mean corpuscular volume, mean corpuscular hemoglobin, mean corpuscular hemoglobin concentration, red cell distribution width, coefficient of variation, platelet count, platelet crit, mean platelet volume, platelet distribution width, coefficient of variation. For this particular study the following parameters were analyzed and compared: Total white blood cell counts (WBC, 10^9^/L), lymphocytes (LYM, 10^9^/L), monocytes (MON, 10^9^/L), neutrophils (NEU, 10^9^/L), hemoglobin (HBG, g/dl), red blood cell counts (RBC, 10^12^/L), platelets (PLT, 10^9^/l), Mean Platelet Volume (MPV, fL) and Platelet Distribution Width (PDW, fL).

At the end of the study, 300 µL of blood samples were collected retro-orbitally in lithium heparin evacuated specimen collection tubes and centrifuged to separate plasma. 100 µL of plasma was dispensed into the VetScan Reagent Rotors and counted in VetScan VS2 Chemistry Analyzer (Allied Analytic, Tampa, FL, USA) to check liver and kidney function parameters. Specifically, we investigated the values of creatinine (CRE, umol/L), blood urea nitrogen (BUN, mmol/L), total bilirubin (TBIL, umol/L), alkaline phosphatase (ALP, U/L), alanine transaminase (ALT, U/L), aspartate aminotransferase (AST, U/L), globulin (GLOB, g/L), albumin (ALB, g/L) and total protein (TP, g/L).

### Statistical analysis

Data are presented as mean ± SEM if not specified otherwise in figure legends. Statistical significance was calculated with a one-way ANOVA test corrected for multiple comparisons (Dunnett’s multiple comparison test), comparing each time point to the start of treatment for all groups. The data of the in vivo studies were analyzed using GraphPad Prism software (version 9). A *p-value < 0.05 is considered as the criterion for statistical significance and is* marked in the figures (* *p* < 0.05, ** *p* < 0.01, *** *p* < 0.001, **** *p* < 0.0001). All the VS2 data was compared to the range the Animal Research Committee of the University of California, Los Angeles (UCLA) established for male C57BL/6 or severe combined immunodeficiency mice.

## Results

### Tumor volume

**Fig. 1:**
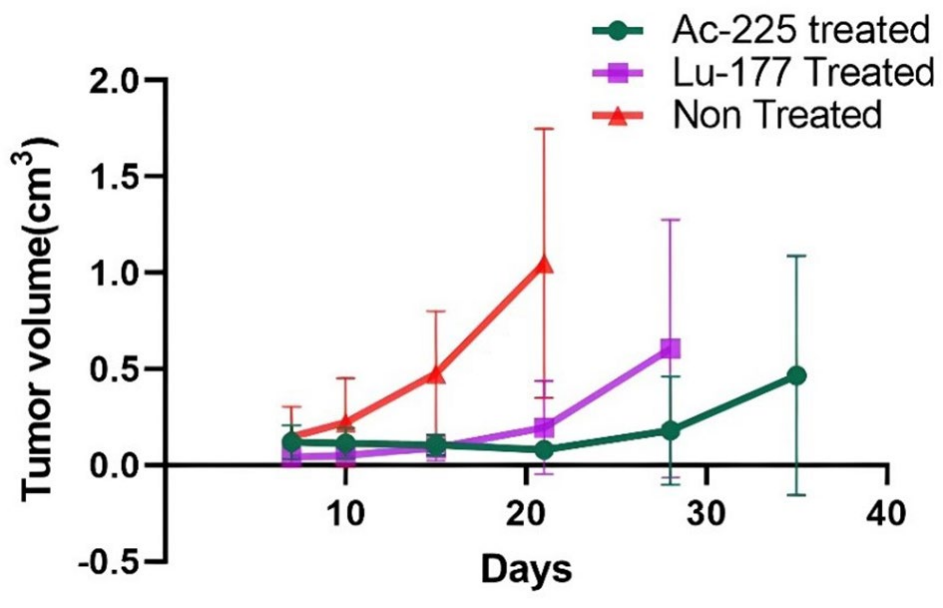
Progression of the tumor by measuring tumor volume from day 6 (before the start of treatment) until the end of the study for all 3 tumor groups

Tumor growth was slower in both groups that received [^225^Ac]Ac-PSMA-617 and [^177^Lu]Lu-PSMA-617 compared to the control group. The group receiving treatment with [^225^Ac]Ac-PSMA-617 showed slower tumor growth and better survival than the group that received [^177^Lu]Lu-PSMA-617 treatment (Fig. 1).

## White blood cells

**Fig. 2:**
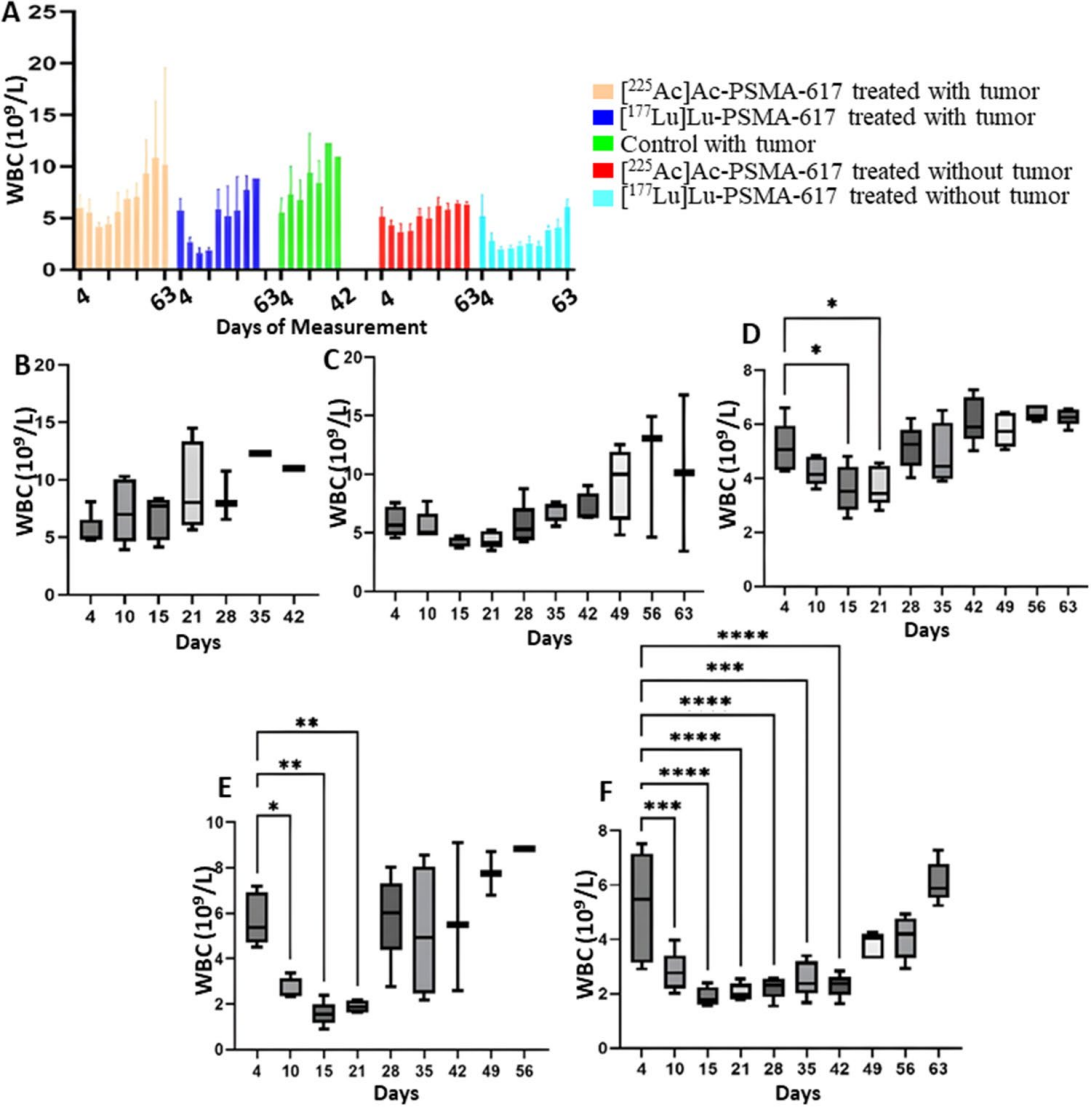
WBC count (**A**) throughout the study for all 5 groups. (**B**) Control with tumor, (**C**) [225Ac]Ac-PSMA-617 treated with tumor, (**D**) [225Ac]Ac-PSMA-617 treated without tumor, (**E**) [177Lu]Lu-PSMA-617 treated with tumor, (**F**)[177Lu]Lu-PSMA-617 treated without tumor show one-way ANOVA test corrected for multiple comparisons (Dunnett’s multiple comparison test), comparing each time point to the first time point (before start of treatment). (**B**) represents WBC analysis for the control group that did not receive treatment, (**C**) and (**D**) for the group that was treated with [225Ac]Ac-PSMA-617 with and without tumor, respectively, (**E**) and (**F**) for the group that was treated with [177Lu]Lu-PSMA-617with and without tumor respectively

**Fig. 3:**
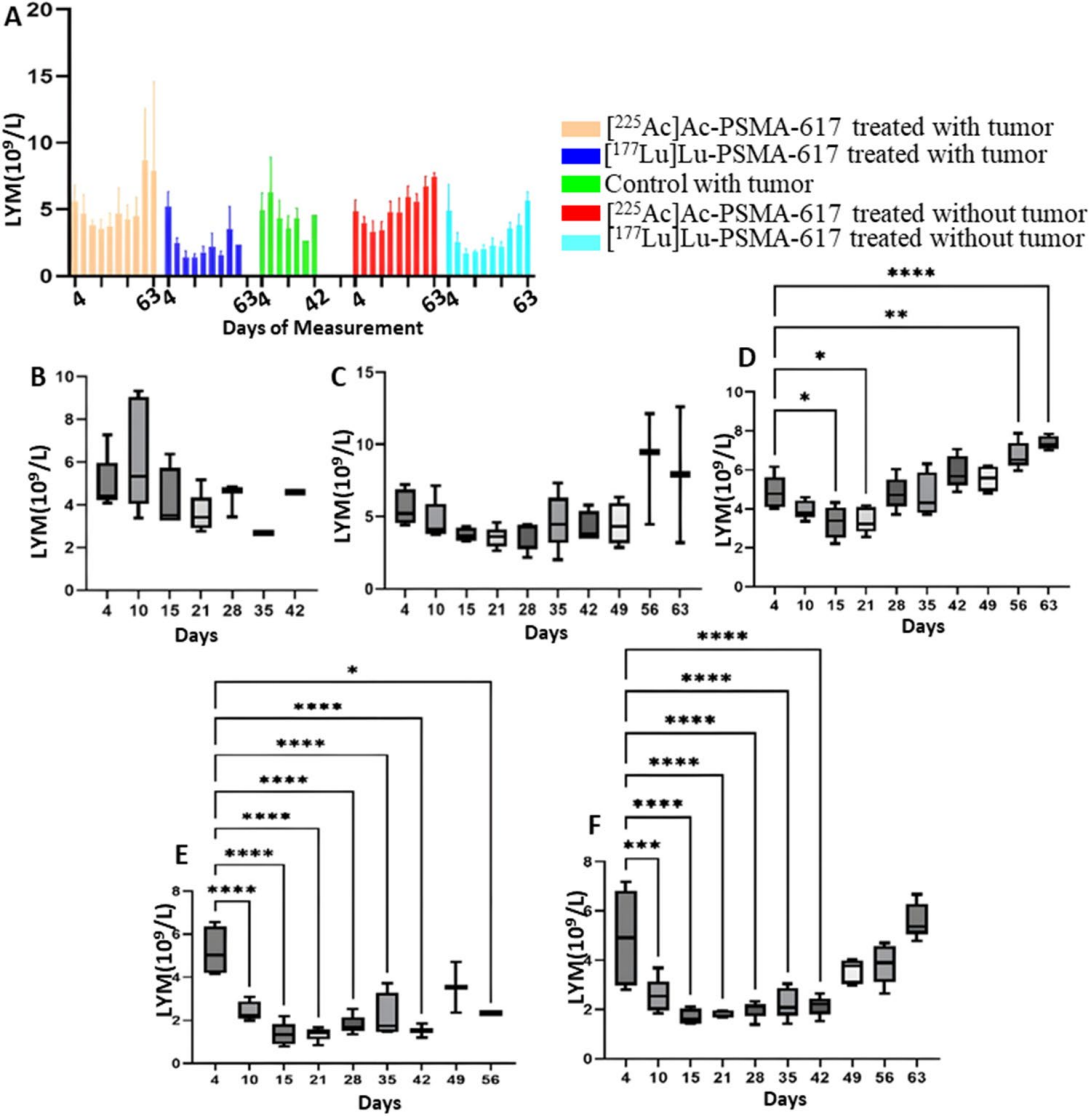
Lymphocyte count (**A**) throughout the study for all 5 groups. (**B**) Control with tumor, (**C**) [^225^Ac]Ac-PSMA-617 treated with tumor, (**D**)[^225^Ac]Ac-PSMA-617 treated without tumor, (**E**) [^177^Lu]Lu-PSMA-617 treated with tumor, (**F**)[^177^Lu]Lu-PSMA-617 treated without tumor show one-way ANOVA test corrected for multiple comparisons (Dunnett’s multiple comparison test), comparing each time point to the first time point (before start of treatment). (**B**) represents lymphocyte analysis for the control group that did not receive treatment, (**C**) and (**D**) for the group that was treated with [^225^Ac]Ac-PSMA-617 with and without tumor, respectively, (**E**) and (**F**) for the group that was treated with [^177^Lu]Lu-PSMA-617with and without tumor respectively

**Fig. 4:**
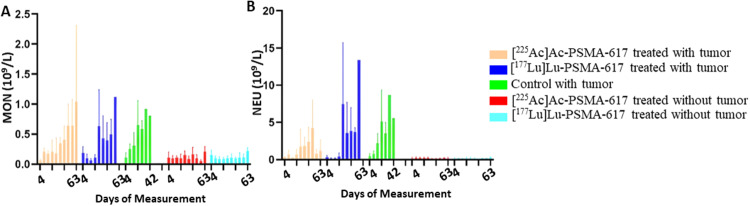
Monocyte count (**A**) and Neutrophil count (**B**) for all five groups over the course of the study

After 3 days post injection of [^177^Lu]Lu-PSMA-617, the WBC count was significantly lower, notably the lymphocytes, compared to [^225^Ac]Ac-PSMA-617. The mean WBC count decreased to a minimum of 1.9 × 10^9^/L from 5.1 × 10^9^/L in the [^177^Lu]Lu-PSMA-617 treated group with tumors and to 1.96 × 10^9^/L from 5.7 × 10^9^/L in the treated group without tumors, 8 days after the treatment. Similarly, the mean lymphocytes most significant drop from 4.9 × 10^9^/L to 1.9 × 10^9^/L and 4.8 × 10^9^/L to 1.7 × 10^9^/L in the [^177^Lu]Lu-PSMA-617 treated groups with and without tumor respectively was seen 8 days after treatment. In the [^225^Ac]Ac-PSMA-617 treated groups, the average WBC count decreased to a minimum of 4.1 × 10^9^/L from initial levels of 5.5 × 10^9^/L and to 3.2 × 10^9^/L from 5 × 10^9^/L with tumor and without tumor respectively 8 days post-treatment. Lymphocyte counts decreased to 4.1 × 10^9^/L from 5.1 × 10^9^/L, and to 3.6 × 10^9^/L from 4.8 × 10^9^/L, in the presence and absence of tumors, respectively, by day 14 post-treatment. [^177^Lu]Lu-PSMA-617 groups took longer for the WBC and lymphocyte counts to recover compared to [^225^Ac]Ac-PSMA-617. At 42, 49, and 56 days, there were no significant differences in the cell count before receiving [^177^Lu]Lu-PSMA-617, suggesting no long-lasting hematotoxicity (Figs. 2 and 3). Monocytes and neutrophils were significantly higher in the tumor groups compared to the non-tumor groups (Fig. 4).

## Red blood cells

**Fig. 5:**
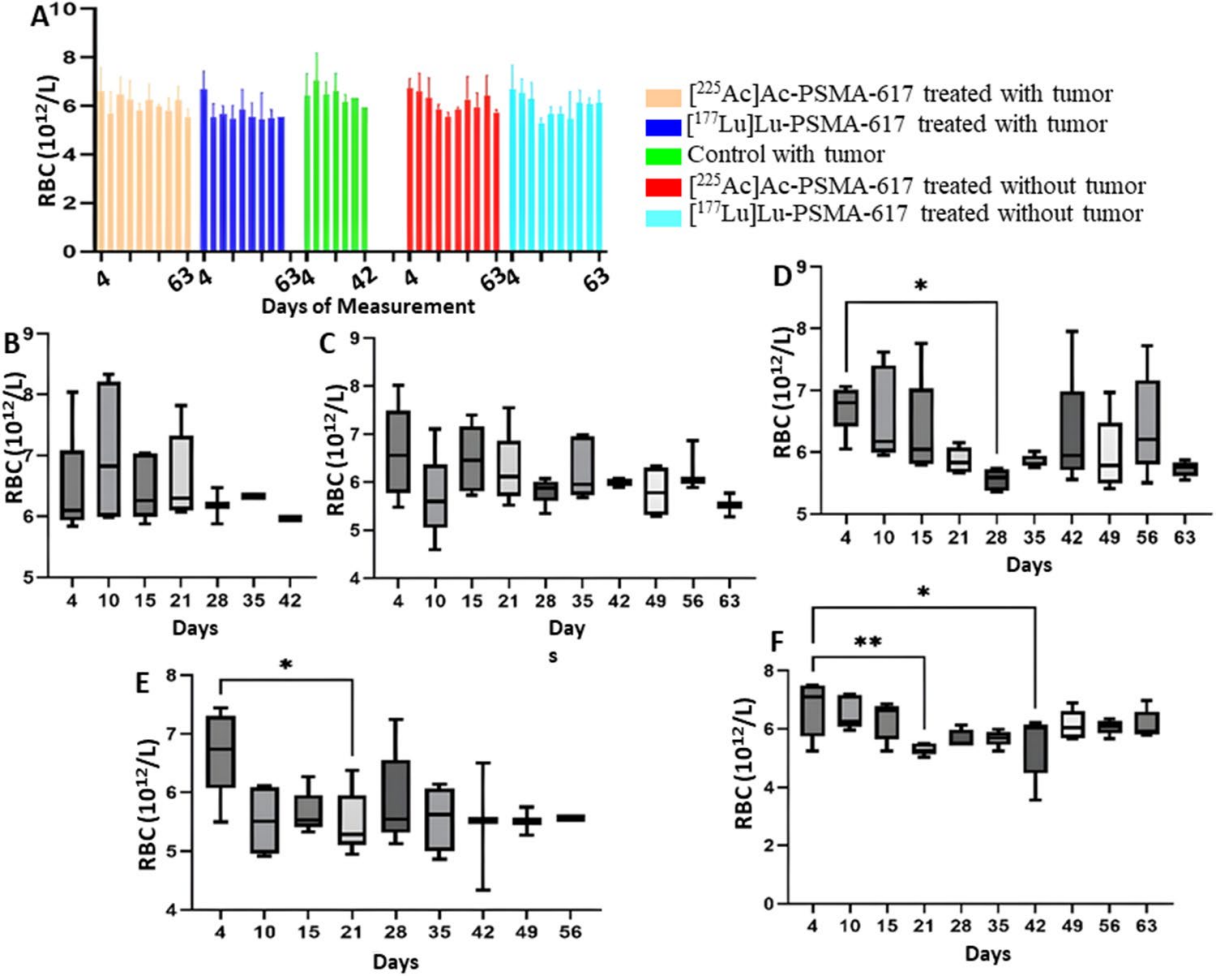
RBC count (**A**) throughout the study for all 5 groups. (**B**) Control with tumor, (**C**)[^225^Ac]Ac-PSMA-617 treated with tumor, (**D**)[^225^Ac]Ac-PSMA-617 treated without tumor, (**E**)[^177^Lu]Lu-PSMA-617 treated with tumor, (**F**)[^177^Lu]Lu-PSMA-617 treated without tumor show one-way ANOVA test corrected for multiple comparisons (Dunnett’s multiple comparison test), comparing each time point to the first time point (before start of treatment). (**B**) represents RBC analysis for the control group that did not receive treatment, (**C**) and (**D**) for the group that was treated with [^225^Ac]Ac-PSMA-617with and without tumor, respectively, (**E**) and (**F**) for the group that was treated with [^177^Lu]Lu-PSMA-617with and without tumor respectively

**Fig. 6:**
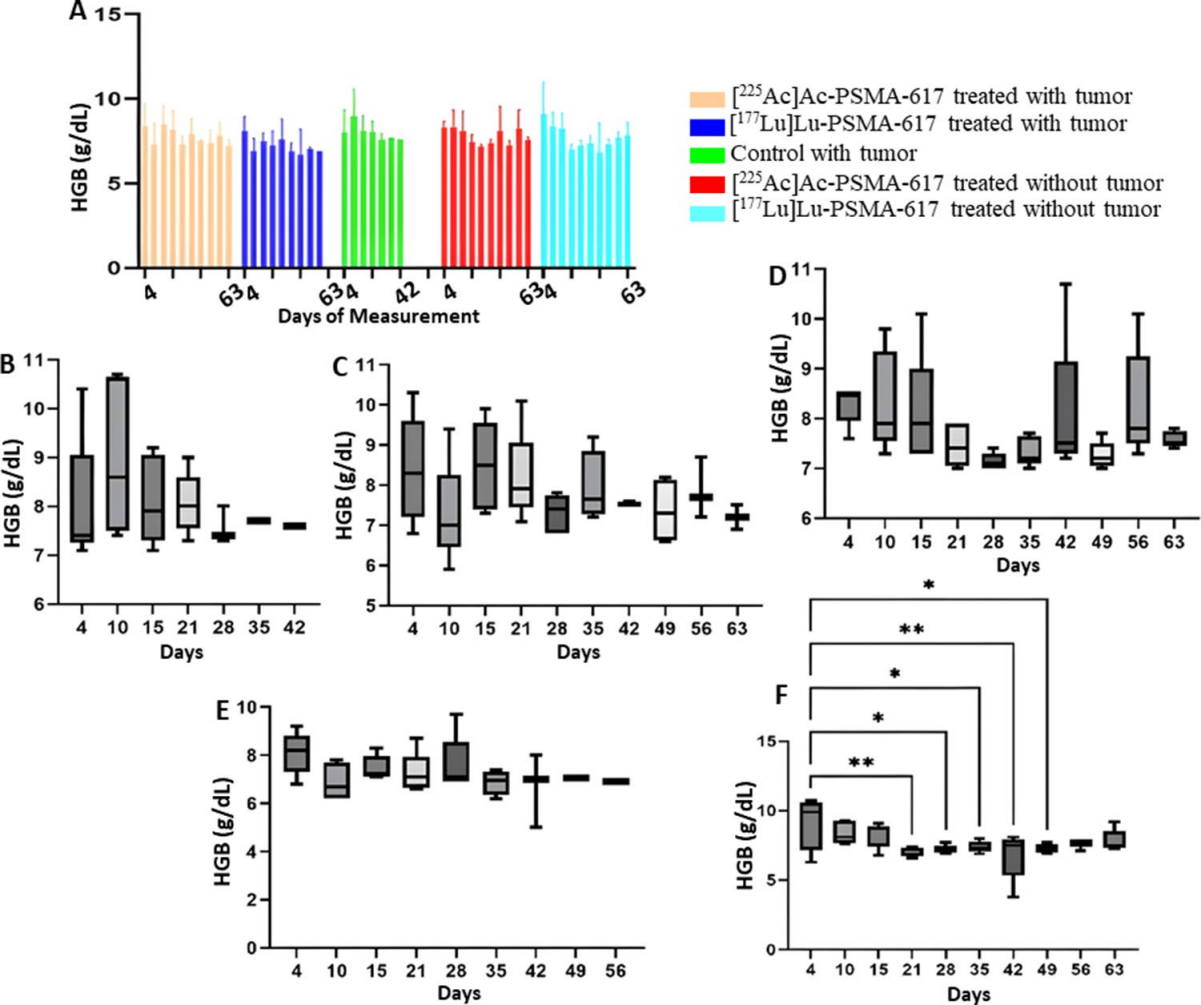
Hemoglobin levels (**A**) for the study for all 5 groups. (**B**) Control with tumor, (**C**)[^225^Ac]Ac-PSMA-617 treated with tumor, (**D**)[^225^Ac]Ac-PSMA-617 treated without tumor, (**E**)[^177^Lu]Lu-PSMA-617 treated with tumor, (**F**)[^177^Lu]Lu-PSMA-617 treated without tumor show one-way ANOVA test corrected for multiple comparisons (Dunnett’s multiple comparison test), comparing each time point to the first time point (before start of treatment). (**B**) represents hemoglobin levels for the control group that did not receive treatment, (**C**) and (**D**) for the group that was treated with [^225^Ac]Ac-PSMA-617 with and without tumor, respectively, (**E**) and (**F**) for the group that was treated with [^177^Lu]Lu-PSMA-617 with and without tumor respectively

A drop was seen in the RBC count on day 21 and day 28 in the [^177^Lu]Lu-PSMA-617 group and the [^225^Ac]Ac-PSMA-617 group without tumor, respectively. The mean RBC count decreased to a minimum of 5.2 × 10^12^/L from 6.7 × 10^12^/L and to 5.0 × 10^12^/L from 7.0 × 10^12^/L in the [^177^Lu]Lu-PSMA-617 treated groups with and without tumor respectively 14 days after treatment. No significant difference in the hemoglobin values was observed except for [^177^Lu]Lu-PSMA-617 group without tumor where the value decrease from 6.9 g/dL to 5.6 g/dL 14 days after treatment (Figs. 5 and 6).

### Platelets

**Fig. 7:**
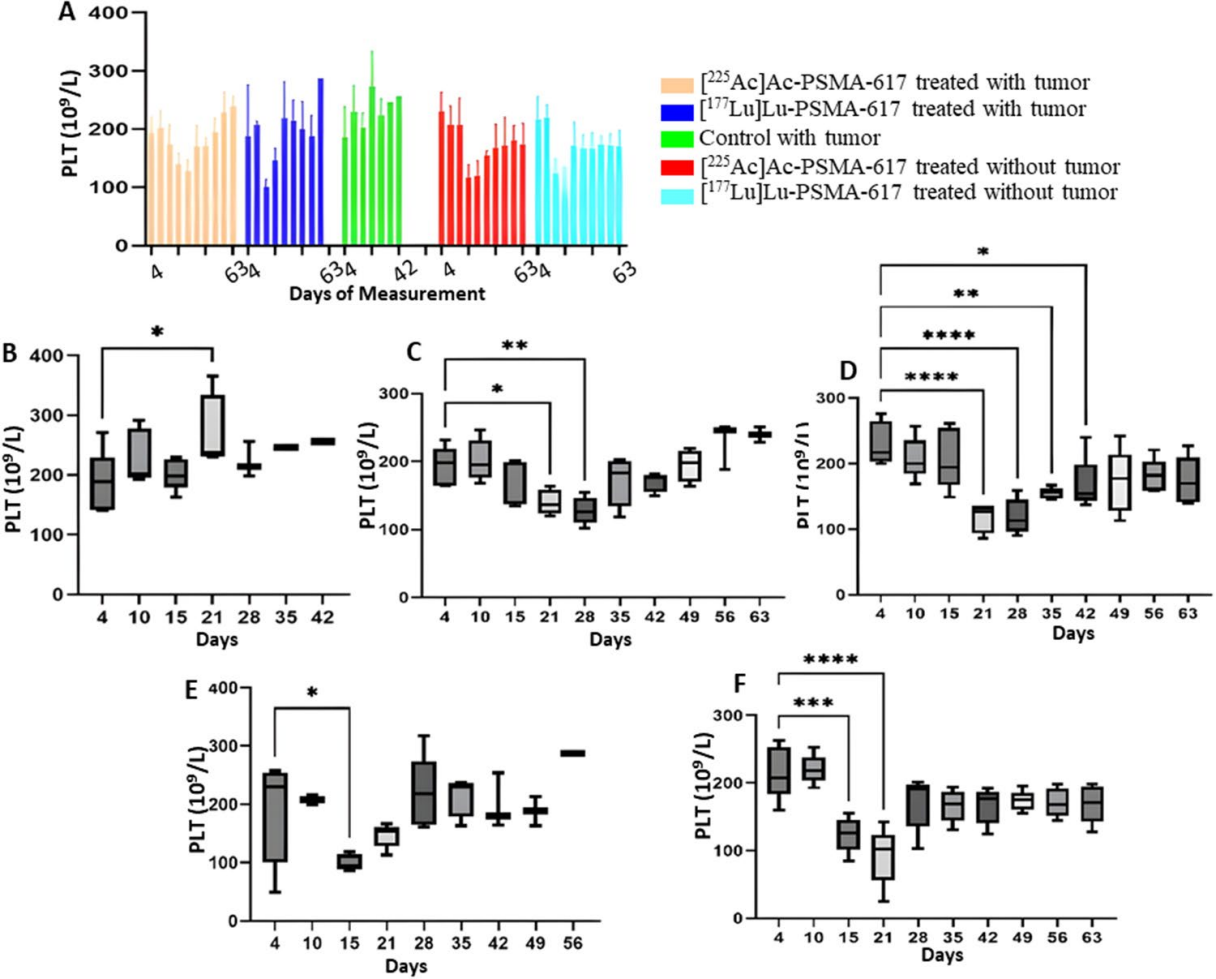
Platelet count (**A**) for the study for all 5 groups. (**B**) Control with tumor, (**C**)[^225^Ac]Ac-PSMA-617 treated with tumor, (**D**)[^225^Ac]Ac-PSMA-617 treated without tumor, (**E**)[^177^Lu]Lu-PSMA-617 treated with tumor, (**F**)[^177^Lu]Lu-PSMA-617 treated without tumor show one-way ANOVA test corrected for multiple comparisons (Dunnett’s multiple comparison test), comparing each time point to the first time point (before start of treatment). (**B**) represents platelet count analysis for the control group that did not receive treatment, (**C**) and (**D**) for the group that was treated with [^225^Ac]Ac-PSMA-617 with and without tumor, respectively, (**E**) and (**F**) for the group that was treated with [^177^Lu]Lu-PSMA-617 with and without tumor respectively

**Fig. 8:**
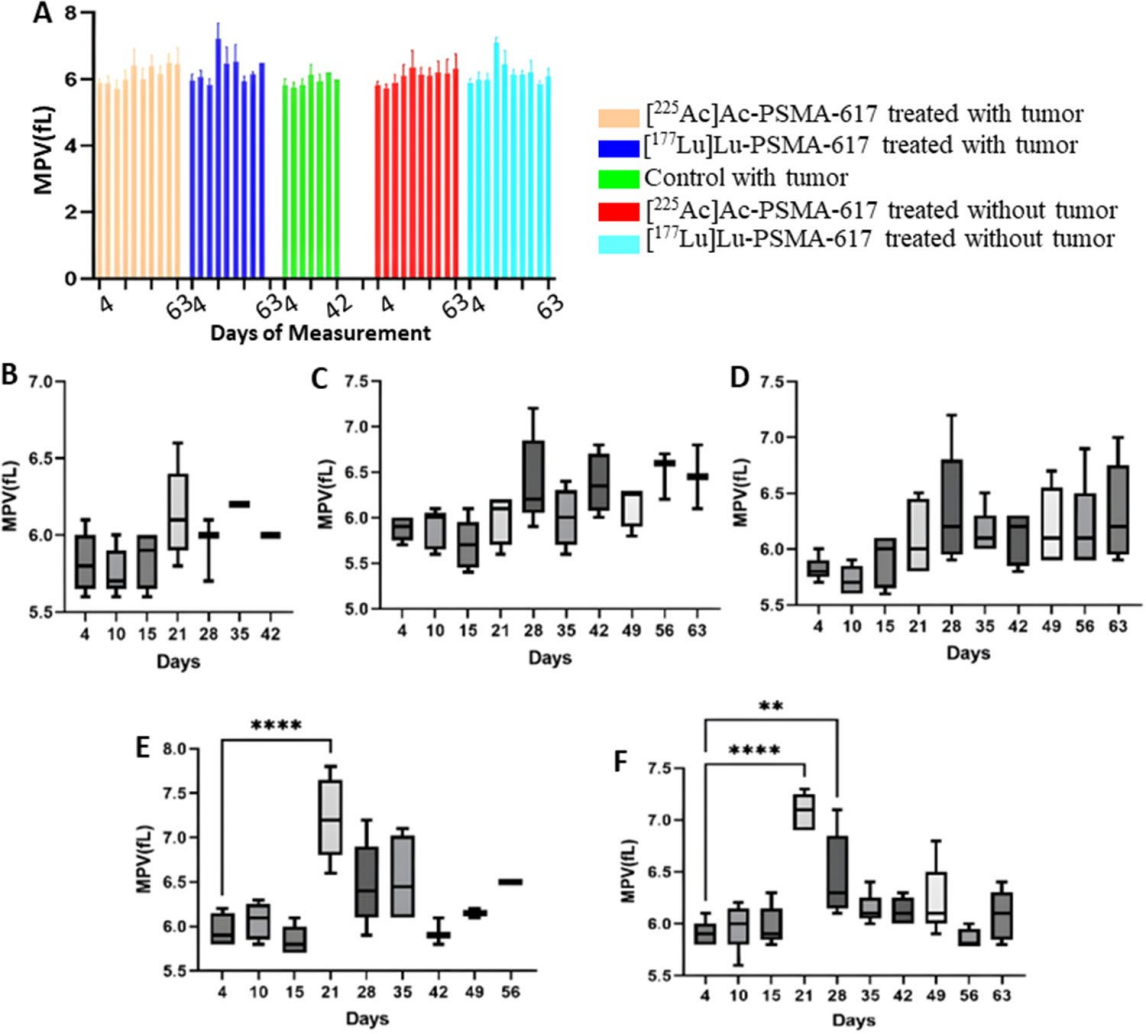
Mean platelet volume (**A**) for the study for all 5 groups. (**B**)Control with tumor, (**C**)[^225^Ac]Ac-PSMA-617 treated with tumor, (**D**)[^225^Ac]Ac-PSMA-617 treated without tumor, (**E**)[^177^Lu]Lu-PSMA-617 treated with tumor, (**F**)[^177^Lu]Lu-PSMA-617 treated without tumor show one-way ANOVA test corrected for multiple comparisons (Dunnett’s multiple comparison test), comparing each time point to the first time point (before start of treatment). (**B**) represents MPV analysis for the control group that did not receive treatment, (**C**) and (**D**) for the group that was treated with [^225^Ac]Ac-PSMA-617 with and without tumor, respectively, (**E**) and (**F**) for the group that was treated with [^177^Lu]Lu-PSMA-617 with and without tumor respectively

**Fig. 9:**
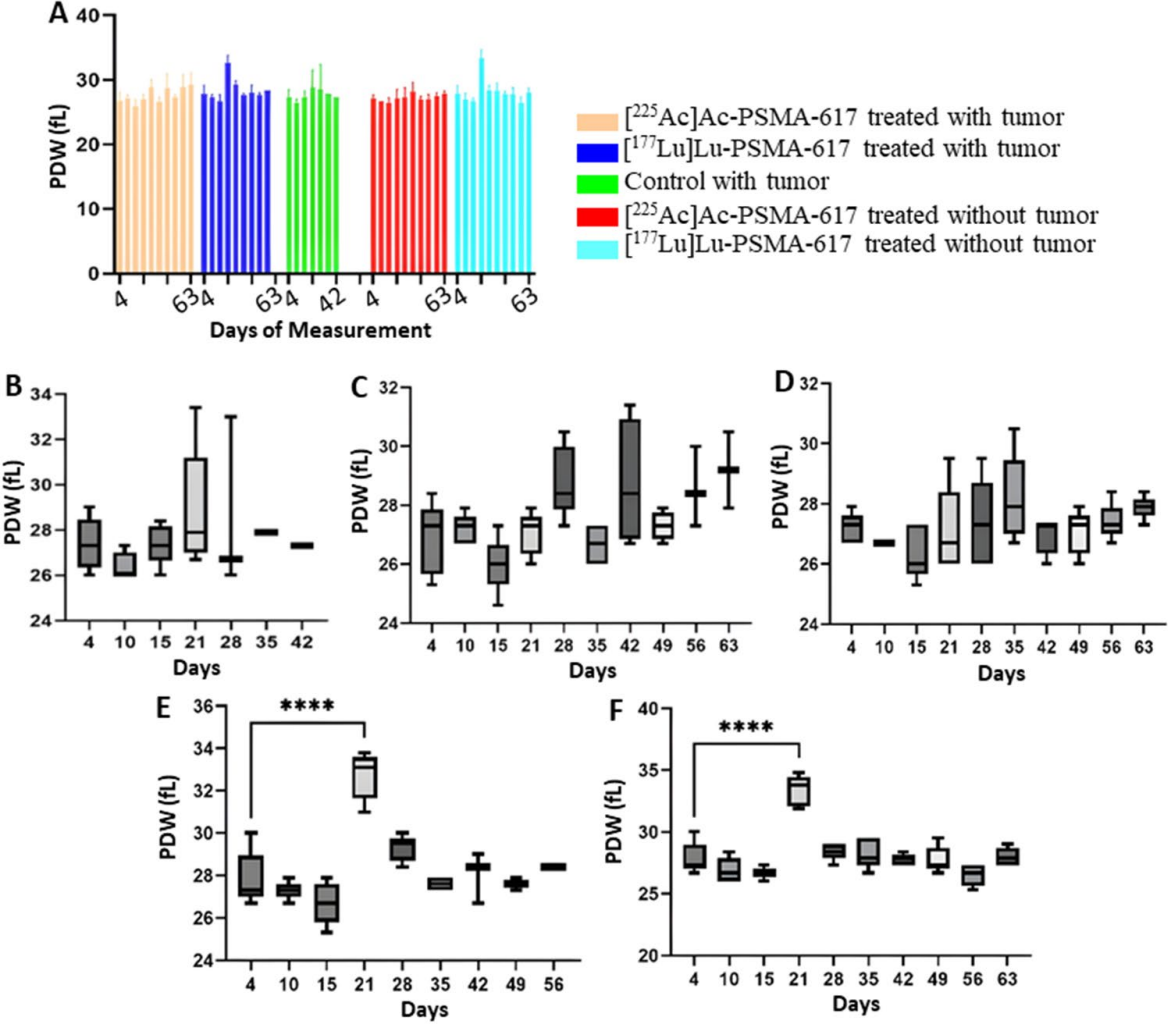
Platelet distribution width (**A**) For the study for all 5 groups. (**B**)Control with tumor, (**C**)[^225^Ac]Ac-PSMA-617 treated with tumor, (**D**)[^225^Ac]Ac-PSMA-617 treated without tumor, (**E**)[^177^Lu]Lu-PSMA-617 treated with tumor, (**F**)[^177^Lu]Lu-PSMA-617 treated without tumor show one-way ANOVA test corrected for multiple comparisons (Dunnett’s multiple comparison test), comparing each time point to the first time point (before start of treatment). (**B**) represents PDW analysis for the control group that did not receive treatment, (**C**) and (**D**) for the group that was treated with [^225^Ac]Ac-PSMA-617 with and without tumor, respectively, (**E**) and (**F**) for the group that was treated with [^177^Lu]Lu-PSMA-617 with and without tumor respectively

From day 21 onwards, a significant drop was seen in the platelet count in the [^225^Ac]Ac-PSMA-617 groups and on day 15 in both [^177^Lu]Lu-PSMA-617 groups. The mean platelet count decreased to a minimum of 100 × 10^9^/L from 210 × 10^9^/L, 8 days post-treatment and to 100 × 10^9^/L from 205 × 10^9^/L, 14 days post-treatment in the [^177^Lu]Lu-PSMA-617 treated groups with and without tumor, respectively. In the [^225^Ac]Ac-PSMA-617 treated groups, the average platelet count decreased to a minimum of 110 × 10^9^/L from initial levels of 200 × 10^9^/L, and to 105 × 10^9^/L from 210 × 10^9^/L with tumor and without tumor, respectively 21 days post-treatment. Recovery in platelet count was observed by day 35 in the [^225^Ac]Ac-PSMA-617 tumor group and day 49 in the non-tumor group, whereas for [^177^Lu]Lu-PSMA-617 groups, recovery was seen by day 21 in the tumor group and day 28 in the non-tumor group. There was no significant difference in the mean platelet volume (MPV) and platelet distribution width (PDW) values among all the groups except [^177^Lu]Lu-PSMA-617 groups on day 21 (14 days post-treatment) where the mean MPV value increased from 5.8fL to 7.1fL and from 5.9fL to 7.2 fl. in the tumor group and non tumor group respectively. Also, the PDW value showed a similar trend where the mean PDW value increased from 27fL to 33.5fL and from 27fL to 34fL in the tumor group and non-tumor group, respectively (Figs. 7, 8 and 9).

### Kidney function tests and liver function tests

**Fig. 10:**
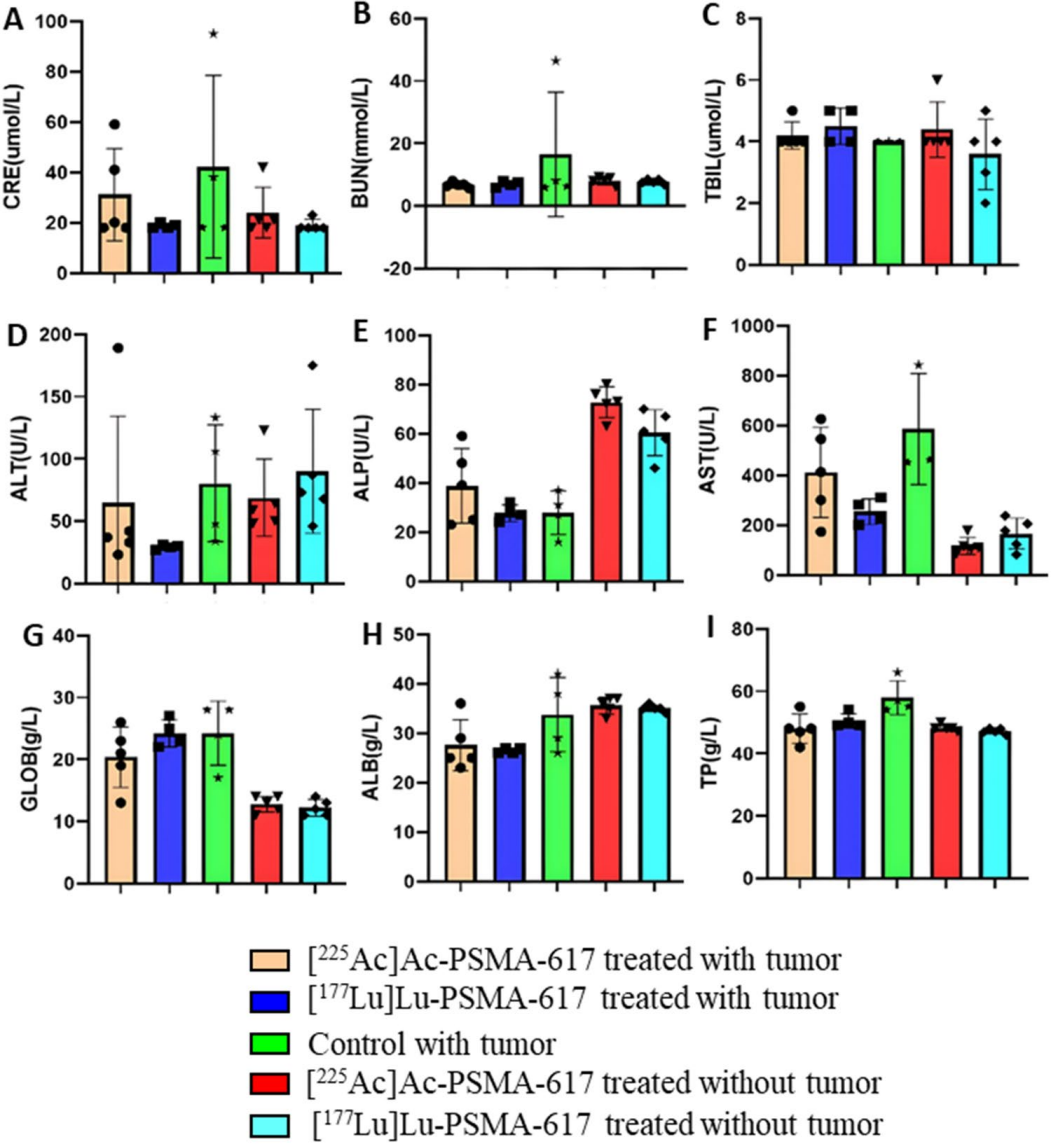
Results of kidney function test and liver function test performed at the end of the study for all groups include the following parameters: (**A**) Creatinine, (**B**) Blood Urea Nitrogen, (**C**) Total Bilirubin, (**D**) Alanine Transaminase, (**E**) Alkaline Phosphatase, (**F**) Aspartate Aminotransferase, (**G**) Globulin, (**H**) Albumin, (**I**) Total Protein

Creatinine, ALP, albumin, and total protein levels were within the reference range for all experimental groups. However, BUN levels were slightly elevated across all groups, with the highest increase observed in the control group. The average ALT values remained within the reference range for all groups, except for one animal each in the [^225^Ac]Ac-PSMA-617 treated group with a tumor and the [^177^Lu]Lu-PSMA-617 treated group without a tumor, both showing elevated values. AST levels were elevated in all tumor groups but remained within the reference range in the [^225^Ac]Ac-PSMA-617 and [^177^Lu]Lu-PSMA-617 treated groups without tumors (Fig. 10). The Animal Research Committee had not provided reference ranges for total bilirubin and globulin levels (Table 2).

**Table 2:** Reference ranges of test parameters

Test Names	Unit	Reference Ranges
ALP (Alkaline phosphatase)	U/L	12–200
ALT (Alanine aminotrasferase)	U/L	22–133
AST (Aspartate aminotrasferase)	U/L	46–221
BUN (Blood urea nitrogen)	mmol/L	0.11–3.94
CRE (Creatinine)	µmol/L	8.84-159.16
ALB (Albumin)	g/L	26–54
TP (Total protein)	g/L	46–73

Reference ranges of test parameters by Animal Research Committee of the University of California, Los Angeles (UCLA) established for C57BL/6 mice

## Discussion

In patients with mCRPC, PSMA-RLT has shown promising results (Rahbar et al. 2017; Alam et al. 2022; Arbuznikova et al. 2023a, b; Fallah et al. 2023; Belli et al. 2020; Burgard et al. 2023; Kratochwil et al. 2016a, b). Response and safety of PSMA-RLT using urea-based molecules showed the potential to expand therapeutic opportunities for metastatic CRPC (Eyben et al. 2020). Treatment outcomes may be enhanced through strategies such as increased activity, dose schedule, and regimen, or combination with other therapeutic modalities, such as radiosensitizer, immunotherapies, etc. (Arbuznikova et al. 2023a, b). In the VISION trial, 831 patients with metastatic castration-resistant prostate cancer were randomized to receive either 177Lu-PSMA-617 plus standard care or standard care alone. The study demonstrated significant improvements in overall survival (15.3 months vs. 11.3 months), image-based progression-free survival, and substantial reductions in PSA levels were noted with [^177^Lu]Lu-PSMA-617 (Rohith 2021). Similarly, Heck and colleagues reported response and safety of [^177^Lu]Lu-PSMA-I&T with over 30% decline of serum PSA levels in 47% of patients and progression-free survival of 4.1 months (Heck et al. 2019). For Ac-225, Kratochwil and colleagues reported on the response and safety of [^225^Ac]-Ac-PSMA-617 in metastatic CRPC patients. A significant decrease in PSA levels was observed without any hematologic issues, although xerostomia emerged as a primary side effect in most patients (Kratochwil et al. 2016a, b, 2018). Preclinical and clinical studies comparing the efficacy of [^225^Ac]Ac-PSMA-617 to [^177^Lu]Lu-PSMA-617 demonstrate a demonstrated better overall survival and progression-free survival than [^177^Lu]Lu-PSMA-617 therapy (Sathekge et al. 2020; Feuerecker et al. 2021). However, despite the numerous reports and exciting progress in using PSMA inhibitors as either α- or β- radiotherapeutics in PC, toxicity in blood, bone marrow, and kidneys remains one of the limiting factors. In this study, we investigated the performance and associated toxicity of [^177^Lu]Lu-PSMA-617 and [^225^Ac]Ac-PSMA-617 in RM1-PGLS, a reliable immunocompetent PSMA-positive PC murine model that also accounts for antitumor immune response (Fendler et al. 2017). Unlike immunodeficient models, syngeneic models use mice with an intact immune system, providing a more accurate representation of the interaction between the immune system and RLT. These models preserve the complexity of the tumor microenvironment, including stromal and immune cells, which is crucial for evaluating the efficacy and potential side effects of radioligand therapies. They involve mice of the same genetic background, reducing variability and increasing experimental results’ reliability. Although imperfect, syngeneic models provide a more realistic context for studying cancer biology and treatment responses, offering insights that are translatable to human clinical settings (Wang et al. 2022).

This study used comparable activities of [^177^Lu]Lu-PSMA-617 and [^225^Ac]Ac-PSMA-617 to evaluate toxicity and treatment response. A 1,000 ratio of activities is typically proposed for these radionuclides to give comparable effects in terms of toxicity due to differences in physical and biological properties, as supported by previous studies (Kratochwil et al. 2017; Belli et al. 2020). However, this ratio may depend on the model used, and determining the maximum tolerated doses for each radionuclide before assessing toxicity in detail is essential. We based our study design on these recommendations, as highlighted by prior work (Fendler et al. 2017; Meyer et al. 2023). One limitation of the study is that these activity levels may not be sufficient to thoroughly investigate renal toxicity, as doses higher than those used here have been shown to cause significant effects in other studies. For example, Kristiansson et al. concluded that hematotoxicity resulting from doses up to 200 MBq of [^177^Lu]Lu-PSMA-617 in a BALB/c mouse model is transient, with normalization within a month (Kristiansson et al. 2022).

Additionally, while the doses administered in this study are below the absorbed dose observed in the kidneys during clinical settings (typically below 10 Gy), further dose-escalation experiments are needed to evaluate renal toxicity at clinically relevant activity levels (Sjogreen Gleisner et al. 2022). Because this study focuses on the effects on the bone marrow, liver, and kidneys from [^225^Ac]Ac-PSMA-617 and [^177^Lu]Lu-PSMA-617, we ensured that the injected solution did not include any free Ac-225 or Lu-177, which primarily distributed to the liver and bone in rodents respectively (Jiang et al. 2018; Hakimi et al. 2015). As expected, [^225^Ac]Ac-PSMA-617 and [^177^Lu]Lu-PSMA-617 treated had slower tumor progression and more prolonged survival than the control group. Noteworthy is that [^225^Ac]Ac-PSMA-617 treatment resulted in improved survival and slower tumor progression than [^177^Lu]Lu-PSMA-617.

Both [^177^Lu]Lu-PSMA-617 and [^225^Ac]Ac-PSMA-617 RLT caused a reduction of WBCs (mainly lymphocytes), RBCs, and platelets, and the decrease was more significant in mice treated with [177Lu]Lu-PSMA-617; however, the values normalized for all the groups within 42 days post-injection. The monocyte and neutrophil count increased in tumor groups, likely due to inflammation in the TME(Jeong et al. 2019). Hemoglobin levels dropped significantly with [^177^Lu]Lu-PSMA-617 in non-tumor groups, and they normalized within 42 days post injection of [^177^Lu]Lu-PSMA-617, whereas for the mice treated with [^225^Ac]Ac-PSMA-617, the drop in hemoglobin levels was within the acceptable range. In physiological conditions, MPV is inversely proportional to the platelet count (Thompson and Jakubowski 1988). A high PDW means that platelet size varies greatly, which is a clue that there is platelet activation. MPV and PDW are both indicators of platelet activation (Vagdatli et al. 2010). We hypothesize that the significant increase in MPV and PDW in the [^177^Lu]Lu-PSMA-617 treated groups is due to a notable decrease in platelet count compared to the [^225^Ac]Ac-PSMA-617 treated groups, where the drop in platelets was relatively lower. However, blood values stabilized within 35–42 days after treatment, and prolonged hematotoxicity was observed.

As indicated in our analyses, the treatment at this dosage did not affect kidney and liver function parameters. Most parameters, including creatinine, alanine transaminase (ALT), alkaline phosphatase (ALP), total protein, and albumin levels, were within the normal range as recommended by the Animal Research Committee of the University of California, Los Angeles (UCLA) for C57BL/6. However, the values for aspartate aminotransferase (AST) were elevated in [^225^Ac]Ac-PSMA-617, [^177^Lu]Lu-PSMA-617 treated with tumors and the control group as well suggesting abnormalities due to tumor progression and not the treatment as the values were typical for the [^225^Ac]Ac-PSMA-617, [^177^Lu]Lu-PSMA-617 treated groups without tumor (Sun et al. 2024). The cause of elevated BUN is challenging to determine because all groups show a slight increase. Still, it is only borderline elevated, except for the control group, which exhibited the highest deviation from the upper limit of the reference range. The Animal Research Committee of the University of California, Los Angeles (UCLA) did not state the acceptable range for globulin levels and total bilirubin. Nonetheless, the results indicate that all tumor-bearing groups exhibited elevated globulin levels compared to the non-tumor groups treated with [^225^Ac]Ac-PSMA-617 and [^177^Lu]Lu-PSMA-617, suggesting that the increased levels are attributable to the presence of tumors rather than toxicity from radioligand therapy. Studies also show that high globulin levels may reflect increased humoral immune activity caused by autoimmune disease, infections, or cancer (Hashash et al. 2022). In total bilirubin, according to the reference values provided by Charles River Laboratories, all groups in the study demonstrated levels that fell within the acceptable range. This indicates that none of the groups showed elevated total bilirubin levels, suggesting no toxicity across the experimental conditions.

Investigating renal damage at higher doses is essential for understanding the safety profile of [^177^Lu]Lu-PSMA-617 and [^225^Ac]Ac-PSMA-617. The current study focused on assessing the acute therapeutic effects and hematological toxicity at the administered doses. Our findings on the renal safety profile of [^177^Lu]Lu-PSMA-617 and [^225^Ac]Ac-PSMA-617 add to the growing evidence on kidney toxicity associated with radioligand therapies. While we observed minimal renal toxicity within the study’s timeframe, several prior studies have reported more pronounced kidney damage, especially at higher doses or with extended observation periods. Busslinger et al. demonstrated dose-dependent renal toxicity, including tubular degeneration and interstitial fibrosis, in immunocompetent mice treated with [^225^Ac]Ac-PSMA-617 and [^177^Lu]Lu-PSMA-617. This highlights the potential for delayed renal effects, which may not be fully captured in our study’s acute evaluation. Similarly, Tschan et al. documented dose-dependent tubular damage with [^177^Lu]Lu-PSMA-I&T, emphasizing the need for long-term follow-up to understand the progression of renal toxicity better. Their findings, along with those of Busslinger et al., suggest that our study’s relatively lower administered doses may have limited the detection of significant renal damage, particularly histopathological changes (Busslinger et al. 2022; Tschan et al. 2023). De Jong et al. demonstrated that kidney toxicity is primarily driven by accumulating radiolabeled PSMA ligands in proximal tubular cells, leading to radiation-induced tubular degeneration and interstitial fibrosis over time. Their findings underscore the importance of assessing absorbed kidney doses and optimizing dose administration strategies to mitigate toxicity. In their preclinical studies, Müller et al. conducted comprehensive histopathological analyses, revealing clear dose thresholds for renal toxicity. Their work highlighted a progression from mild tubular degeneration at lower doses to significant tubular atrophy and fibrosis at higher doses, reinforcing the concept of dose-dependent toxicity. Importantly, their findings indicated that renal damage often becomes apparent only after a prolonged period, further emphasizing the need for extended observation in preclinical studies to assess long-term safety profiles. The results from de Jong and Müller’s collaborators provide critical context to our findings. While we observed that renal function markers, such as creatinine and BUN, remained within normal ranges, their studies underscore the importance of incorporating long-term follow-up and histopathological evaluations into future research. Our study’s shorter observation period and the relatively low administered doses may account for the lack of observable renal damage in this timeframe. These previous studies collectively emphasize that kidney toxicity may not manifest acutely but evolve over more extended periods, particularly with increased activity levels (Jong et al. 2002, 2005; Müller et al. 2015, 2016). Our findings, in conjunction with those of Busslinger et al., Tschan et al., de Jong et al., and Müller et al., highlight the complex interplay between dose, time, and renal toxicity in PSMA-targeted radioligand therapies. While our results demonstrate the short-term safety of [^177^Lu]Lu-PSMA-617 and [^225^Ac]Ac-PSMA-617 in terms of renal function, further studies are needed to investigate long-term kidney damage, including dose-escalation experiments and detailed histopathological analyses. Future work should also incorporate dosimetry-based approaches to refine activity levels and optimize safety while preserving therapeutic efficacy.

Although an in-depth evaluation of renal toxicity using higher doses would provide valuable insights, conducting such experiments is challenging in preclinical models due to animal welfare considerations. The associated ethical concerns, particularly in tumor-bearing animals with significant morbidity, limit the practicality of long-term studies or repeated high-dose exposure. Future research may explore alternative approaches to address this limitation while adhering to humane endpoints. Future studies will include dose-escalation experiments to evaluate potential renal toxicity at clinically relevant doses. We acknowledge that the doses administered in our study differ from those typically used in clinical settings. However, we recognize the importance of conducting additional experiments with dose-adjusted radioactivity levels that align more closely with clinical scenarios to ensure translational relevance. We intend to address this in future studies.

A key limitation of the current study is the lack of analysis of immune cell populations, which would have provided valuable insights into the potential role of immune responses in the therapeutic effects of radioligands. The effectiveness of alpha-emitters against macroscopic tumors may partly result from immune system activation, as suggested by prior studies (Kratochwil et al. 2017). This hypothesis could be explored further using the syngeneic model employed here, as it preserves the complexity of tumor-immune interactions. Including immune profiling and mechanistic investigations in future studies would enhance our understanding of particle-specific mechanisms and their contribution to therapeutic outcomes. Previous studies, such as those by Benard et al. and Babich et al., evaluated PSMA-targeted radioligand therapies in nude mouse prostate cancer xenografts, showing tumor reduction and progression delay (Benard et al. 2020; Babich et al. 2020). However, nude mice lack a functional immune system, limiting the ability to capture immune interactions crucial in human therapies.

In contrast, our use of immunocompetent C57BL/6 RM1-PGLS mice better mimics the human immune system and offers insights into immune-mediated effects and potential toxicities. We observed that [^177^Lu]Lu-PSMA-617 induced more hematotoxicity than [^225^Ac]Ac-PSMA-617, highlighting the importance of immune system interactions in recovery and toxicity. While nude mouse xenografts revealed kidney toxicity, our immunocompetent model suggests that immune system factors may modulate these effects. This demonstrates the added value of immunocompetent mice in accurately predicting clinical outcomes, especially when considering combination therapies with immune modulators.

As the field progresses towards combination therapies, including immune checkpoint inhibitors, including immunocompetent models is crucial for understanding the broader therapeutic context and the full range of immune-mediated effects, making them indispensable for more clinically relevant preclinical evaluations. As exploring the immune microenvironment in the therapeutic effects of radioligands was not the primary focus of the current study, we did not include a detailed analysis of immune cell populations. However, we recognize that alpha and beta particles may differentially influence the tumor microenvironment and immune cells. Incorporation of flow cytometry data or immunohistochemical analyses to compare immune cell populations (e.g., T cells, macrophages, and other immune modulators) between treatment groups in future studies, along with investigating DNA damage response and tumor microenvironment remodeling induced by each radioligand would provide valuable insights into the role of immune cells in mediating therapeutic responses and help delineate the contribution of particle-specific mechanisms to therapeutic outcomes. We acknowledge that clinical studies have previously documented the toxicity of [^177^Lu]Lu-PSMA-617 and [^225^Ac]Ac-PSMA-617. However, this study specifically aims to address the hematotoxicity and recovery dynamics in a controlled preclinical setting using a syngeneic mouse model by employing RM1-PGLS tumor cells in immunocompetent C57BL/6 mice. We have tried to provide a closer approximation to the immune environment than xenograft models in immunodeficient mice, which are commonly used in similar studies. This model allowed us to evaluate transient hematotoxicity and subsequent recovery, providing a detailed timeline that could guide clinical monitoring and management of blood cell recovery following therapy. To further explore the differences in radioactivity levels and their relevance to clinical studies, we aim to conduct comparative analyses using clinically relevant activity levels, incorporating dosimetry models that bridge the gap between preclinical and clinical scales. It would be insightful to include immune profiling and mechanistic investigations to comprehensively understand the interplay between radiation type, dosage, and biological response.

## Conclusion

The hematotoxicity resulting from the infusion of [^225^Ac]Ac-PSMA-617 and [^177^Lu]Lu-PSMA-617 in our mouse model is transient, and mice exhibit normalized blood values within a month with no other apparent adverse effects (e.g., deaths, bleeding, or other abnormalities). The findings suggest that these activities in animal models contribute to a more relevant understanding of renal toxicity and improve the translation of therapeutic strategies from preclinical models to clinical applications. More studies need to assess the effectiveness and side effects of [^225^Ac]Ac-PSMA-617 in relevant PC and mCRPC models.

## Electronic supplementary material

Below is the link to the electronic supplementary material.

Supplementary Material 1

## Data Availability

The datasets generated during and/or analyzed during the current study are available from the corresponding author on reasonable request.

## References

[CR12] Alam MR, et al. A review of 177Lutetium-PSMA and 225Actinium-PSMA as emerging theranostic agents in prostate Cancer. Cureus. 2022;14(9):e29369. 36284803 10.7759/cureus.29369 PMC9584169

[CR13] Arbuznikova D, et al. Towards improving the efficacy of PSMA-Targeting radionuclide therapy for Late-Stage prostate Cancer-Combination strategies. Curr Oncol Rep. 2023a;25(11):1363–74. 37861915 10.1007/s11912-023-01458-6 PMC10640479

[CR22] Arbuznikova D, et al. Towards improving the efficacy of PSMA-Targeting radionuclide therapy for Late-Stage prostate Cancer-Combination strategies. Current Oncology Reports; 2023b. 10.1007/s11912-023-01458-6 PMC10640479 37861915

[CR48] Babich JW, et al. Therapeutic efficacy of PSMA-Targeted radioligands in prostate Cancer xenograft models. Eur J Nucl Med Mol Imaging. 2020;47(5):1175–84. 10.1007/s00259-019-04556-7.

[CR2] Beer TM, et al. Enzalutamide in men with Chemotherapy-naive metastatic Castration-resistant prostate cancer: extended analysis of the phase 3 PREVAIL study. Eur Urol. 2017;71(2):151–4. 27477525 10.1016/j.eururo.2016.07.032 PMC5570461

[CR19] Belli ML, et al. Targeted alpha therapy in mCRPC (Metastatic Castration-Resistant prostate Cancer) patients: predictive dosimetry and toxicity modeling of (225)Ac-PSMA (Prostate-Specific membrane Antigen). Front Oncol. 2020;10:531660. 33251129 10.3389/fonc.2020.531660 PMC7674768

[CR47] Benard F, et al. Targeted alpha therapy for prostate Cancer using 177Lu-PSMA-617. J Nucl Med. 2020;61(2):156–63. 10.2967/jnumed.119.235300.

[CR20] Burgard C et al. Tumor sink effect with prostate-Specific membrane Antigen-Targeted theranostics in patients with metastatic Castration-Resistant prostate cancer: Intra-Individual evaluations. Cancers (Basel), 2023. 15(9). 10.3390/cancers15092592 PMC10177482 37174058

[CR41] Busslinger S, et al. Preclinical evaluation of [177Lu]Lu-PSMA-617 and [225Ac]Ac-PSMA-617: therapeutic efficacy and toxicity. Cancers. 2022;14(2):234. DOI: 10.3390/cancers14020234.35008395

[CR6] Calais J, et al. Safety of PSMA-Targeted molecular radioligand therapy with (177)Lu-PSMA-617: results from the prospective multicenter phase 2 trial RESIST-PC (NCT03042312). J Nucl Med. 2021;62(10):1447–56. 34272322 10.2967/jnumed.121.262543 PMC8724902

[CR3] Crawford ED, et al. Treating patients with metastatic castration resistant prostate cancer: A comprehensive review of available therapies. J Urol. 2015;194(6):1537–47. 26196735 10.1016/j.juro.2015.06.106

[CR44] De Jong M, et al. Radiation-induced kidney damage after treatment with radiolabeled peptides. Eur J Nucl Med Mol Imaging. 2002;29(5):641–50. 10.1007/s00259-001-0716-5.11976802

[CR43] De Jong M, et al. Renal toxicity of radiolabeled peptides and antibody fragments: preclinical studies. J Nuclear Med. 2005;46(S1):S164–71.

[CR7] Djaileb L, et al. Presurgical (68)Ga-PSMA-11 positron emission tomography for biochemical recurrence risk assessment: A Follow-up analysis of a multicenter prospective phase 3 imaging trial. Eur Urol. 2023;84(6):588–96. 37482512 10.1016/j.eururo.2023.06.022

[CR14] Fallah J, et al. FDA approval summary: lutetium Lu 177 Vipivotide tetraxetan for patients with metastatic Castration-Resistant prostate Cancer. Clin Cancer Res. 2023;29(9):1651–7. 36469000 10.1158/1078-0432.CCR-22-2875 PMC10159870

[CR29] Fendler WP, et al. Establishing (177)Lu-PSMA-617 radioligand therapy in a syngeneic model of murine prostate Cancer. J Nucl Med. 2017;58(11):1786–92. 28546332 10.2967/jnumed.117.193359 PMC6944167

[CR28] Feuerecker B, et al. Activity and adverse events of Actinium-225-PSMA-617 in advanced metastatic Castration-resistant prostate Cancer after failure of Lutetium-177-PSMA. Eur Urol. 2021;79(3):343–50. 33293081 10.1016/j.eururo.2020.11.013

[CR5] Ghosh A, Heston WD. Tumor target prostate specific membrane antigen (PSMA) and its regulation in prostate cancer. J Cell Biochem. 2004;91(3):528–39. 14755683 10.1002/jcb.10661

[CR11] Groener D, et al. Hematologic safety of (177)Lu-PSMA-617 radioligand therapy in patients with metastatic castration-resistant prostate cancer. EJNMMI Res. 2021;11(1):61. 34216290 10.1186/s13550-021-00805-7 PMC8254689

[CR35] Hakimi A, et al. Production, quality control, biological evaluation and biodistribution modeling of Lutetium-177 maltolate as a viable bone pain palliative in skeletal metastasis. J Radioanal Nucl Chem. 2015;303(1):1–10.

[CR15] Hartrampf PE et al. *Hematotoxicity and Nephrotoxicity in Prostate Cancer Patients Undergoing Radioligand Therapy with [(177)Lu]Lu-PSMA I&T.* Cancers (Basel), 2022. 14(3). 10.3390/cancers14030647 PMC8833540 35158913

[CR40] Hashash JG, et al. Elevated serum Globulin fraction as a biomarker of multiyear disease severity in inflammatory bowel disease. Ann Gastroenterol. 2022;35(6):609–17. 36406970 10.20524/aog.2022.0748 PMC9648529

[CR24] Heck MM, et al. Treatment outcome, toxicity, and predictive factors for radioligand therapy with (177)Lu-PSMA-I&T in metastatic Castration-resistant prostate Cancer. Eur Urol. 2019;75(6):920–6. 30473431 10.1016/j.eururo.2018.11.016

[CR36] Jeong J, Suh Y, Jung K. Context drives diversification of monocytes and neutrophils in orchestrating the tumor microenvironment. Front Immunol. 2019;10:1817. 31474975 10.3389/fimmu.2019.01817 PMC6706790

[CR34] Jiang Z, et al. In vivo evaluation of free and chelated Accelerator-produced Actinium- 225 - Radiation dosimetry and toxicity results. Curr Radiopharm. 2018;11(3):215–22. 29683101 10.2174/1874471011666180423120707

[CR18] Khreish F, et al. (225)Ac-PSMA-617/(177)Lu-PSMA-617 tandem therapy of metastatic castration-resistant prostate cancer: pilot experience. Eur J Nucl Med Mol Imaging. 2020;47(3):721–8. 31758224 10.1007/s00259-019-04612-0

[CR21] Kratochwil C, et al. PSMA-Targeted radionuclide therapy of metastatic Castration-Resistant prostate Cancer with 177Lu-Labeled PSMA-617. J Nucl Med. 2016a;57(8):1170–6. 26985056 10.2967/jnumed.115.171397

[CR25] Kratochwil C, et al. 225Ac-PSMA-617 for PSMA-Targeted alpha-Radiation therapy of metastatic Castration-Resistant prostate Cancer. J Nucl Med. 2016b;57(12):1941–4. 27390158 10.2967/jnumed.116.178673

[CR17] Kratochwil C, et al. Targeted alpha-Therapy of metastatic Castration-Resistant prostate Cancer with (225)Ac-PSMA-617: dosimetry estimate and empiric dose finding. J Nucl Med. 2017;58(10):1624–31. 28408529 10.2967/jnumed.117.191395

[CR26] Kratochwil C, et al. Targeted alpha-Therapy of metastatic Castration-Resistant prostate Cancer with (225)Ac-PSMA-617: Swimmer-Plot analysis suggests efficacy regarding duration of tumor control. J Nucl Med. 2018;59(5):795–802. 29326358 10.2967/jnumed.117.203539

[CR31] Kristiansson A et al. Hematological toxicity in mice after high activity injections of (177)Lu-PSMA-617. Pharmaceutics, 2022. 14(4). 10.3390/pharmaceutics14040731 PMC9032768 35456565

[CR33] Meyer C, et al. Tandem isotope therapy with (225)Ac- and (177)Lu-PSMA-617 in a murine model of prostate Cancer. J Nucl Med. 2023;64(11):1772–8. 37797974 10.2967/jnumed.123.265433 PMC10626377

[CR46] Müller C, et al. Renal dosimetry and toxicity of radiolabeled PSMA inhibitors in preclinical models. Mol Imaging Biology. 2015;17(6):815–21. 10.1007/s11307-015-0862-3.

[CR45] Müller C, et al. Targeted radioligand therapy with PSMA ligands: preclinical studies on renal toxicity. J Nucl Med. 2016;57(12):1941–7. 10.2967/jnumed.116.177394.27390158

[CR10] Rahbar K, et al. German multicenter study investigating 177Lu-PSMA-617 radioligand therapy in advanced prostate Cancer patients. J Nucl Med. 2017;58(1):85–90. 27765862 10.2967/jnumed.116.183194

[CR23] Rohith G. VISION trial: (177)Lu-PSMA-617 for progressive metastatic castration-resistant prostate cancer. Indian J Urol. 2021;37(4):372–3. 34759536 10.4103/iju.iju_292_21 PMC8555571

[CR16] Sathekge M, et al. (225)Ac-PSMA-617 in chemotherapy-naive patients with advanced prostate cancer: a pilot study. Eur J Nucl Med Mol Imaging. 2019;46(1):129–38. 30232539 10.1007/s00259-018-4167-0 PMC6267694

[CR27] Sathekge M, et al. Predictors of overall and Disease-Free survival in metastatic Castration-Resistant prostate Cancer patients receiving (225)Ac-PSMA-617 radioligand therapy. J Nucl Med. 2020;61(1):62–9. 31101746 10.2967/jnumed.119.229229

[CR32] Sjogreen Gleisner K, et al. EANM dosimetry committee recommendations for dosimetry of 177Lu-labelled somatostatin-receptor- and PSMA-targeting ligands. Eur J Nucl Med Mol Imaging. 2022;49(6):1778–809. 35284969 10.1007/s00259-022-05727-7 PMC9015994

[CR39] Sun G, et al. Metabolomics reveals ascorbic acid inhibits ferroptosis in hepatocytes and boosts the effectiveness of anti-PD1 immunotherapy in hepatocellular carcinoma. Cancer Cell Int. 2024;24(1):192. 38822322 10.1186/s12935-024-03342-0 PMC11143590

[CR1] Sung H, et al. Global Cancer statistics 2020: GLOBOCAN estimates of incidence and mortality worldwide for 36 cancers in 185 countries. CA Cancer J Clin. 2021;71(3):209–49. 33538338 10.3322/caac.21660

[CR4] Tateishi U. Prostate-specific membrane antigen (PSMA)-ligand positron emission tomography and radioligand therapy (RLT) of prostate cancer. Jpn J Clin Oncol. 2020;50(4):349–56. 32147685 10.1093/jjco/hyaa004 PMC7160915

[CR37] Thompson CB, Jakubowski JA. The pathophysiology and clinical relevance of platelet heterogeneity. Blood. 1988;72(1):1–8. 3291975

[CR42] Tschan V, et al. Dose-dependent kidney toxicity of [177Lu]Lu-PSMA-I&T in preclinical models and implications for clinical use. J Nucl Med. 2023;64(5):731–40. 10.2967/jnumed.122.264838.36522186

[CR38] Vagdatli E, et al. Platelet distribution width: a simple, practical and specific marker of activation of coagulation. Hippokratia. 2010;14(1):28–32. 20411056 PMC2843567

[CR9] von Eyben FE et al. Optimizing PSMA radioligand therapy for patients with metastatic Castration-Resistant prostate cancer. A systematic review and Meta-Analysis. Int J Mol Sci, 2020. 21(23). 10.3390/ijms21239054 PMC7730994 33260535

[CR30] Wang Y, et al. Preclinical models for development of immune-oncology therapies. Immuno-oncol Insights. 2022;3(8):379–98. 37132013 10.18609/ioi.2022.41 PMC10150782

[CR8] Weiner AB, et al. Impact of PSMA PET on prostate Cancer management. Curr Treat Options Oncol. 2024;25(2):191–205. 38270802 10.1007/s11864-024-01181-9 PMC11034977

