# Supplementary material for: Hematological toxicity of [225Ac]Ac-PSMA-617 and [177Lu]Lu-PSMA-617 in RM1-PGLS syngeneic mouse model
Source: EJNMMI Radiopharm Chem. 2025 Mar 24;10:12. doi: 10.1186/s41181-025-00333-y (PMC11933494; doi:10.1186/s41181-025-00333-y)
Supplement: Supplementary file 1 — Supplementary Material 1 [file 41181_2025_333_MOESM1_ESM.docx]

**-Supplemental Materials-**

**Hematological Toxicity of [^225^Ac]Ac-PSMA-617 and [^177^Lu]Lu-PSMA-617 in RM1-PGLS syngeneic mouse model**

**Meryl Maria Vilangattil^1^, Abir Swaidan^1^, Jonathan Godinez^1^, Marco F. Taddio^1^, Johannes Czernin^1,2^, Christine E. Mona^1,2^ and Giuseppe Carlucci^1,2^**

^1^Ahmanson Translational Theranostics Division, Department of Molecular and Medical Pharmacology, David Geffen School of Medicine, UCLA, Los Angeles, California

^2^Jonsson Comprehensive Cancer Center, UCLA, Los Angeles, California

**Corresponding authors:**

| Giuseppe Carlucci  [GCarlucci@mednet.ucla.edu](mailto:GCarlucci@mednet.ucla.edu)  <https://orcid.org/0000-0001-7494-335X> |
| --- |

Meryl Maria Vilangattil

[merylmaria7@gmail.com](mailto:merylmaria7@gmail.com)

[https://orcid.org/0009-0000-8083-8097](https://eur03.safelinks.protection.outlook.com/?url=https%3A%2F%2Forcid.org%2F0009-0000-8083-8097&data=05%7C01%7Cm.m.s.vilangattil%40umcg.nl%7C50015ea694e341233e8d08dbf279141a%7C335122f9d4f44d67a2fccd6dc20dde70%7C0%7C0%7C638370374055580653%7CUnknown%7CTWFpbGZsb3d8eyJWIjoiMC4wLjAwMDAiLCJQIjoiV2luMzIiLCJBTiI6Ik1haWwiLCJXVCI6Mn0%3D%7C3000%7C%7C%7C&sdata=yfo5YctChq47vCfmCXBTiwUHH1WUKKhuVNEgEZl6bk4%3D&reserved=0)

**Address of the corresponding authors:**

| Ahmanson Translational Theranostics Division, Department of Molecular and Medical Pharmacology  David Geffen School of Medicine, UCLA, Los Angeles, California  650 Charles E Young Dr S, Los Angeles, California 90095, USA |
| --- |

Counts


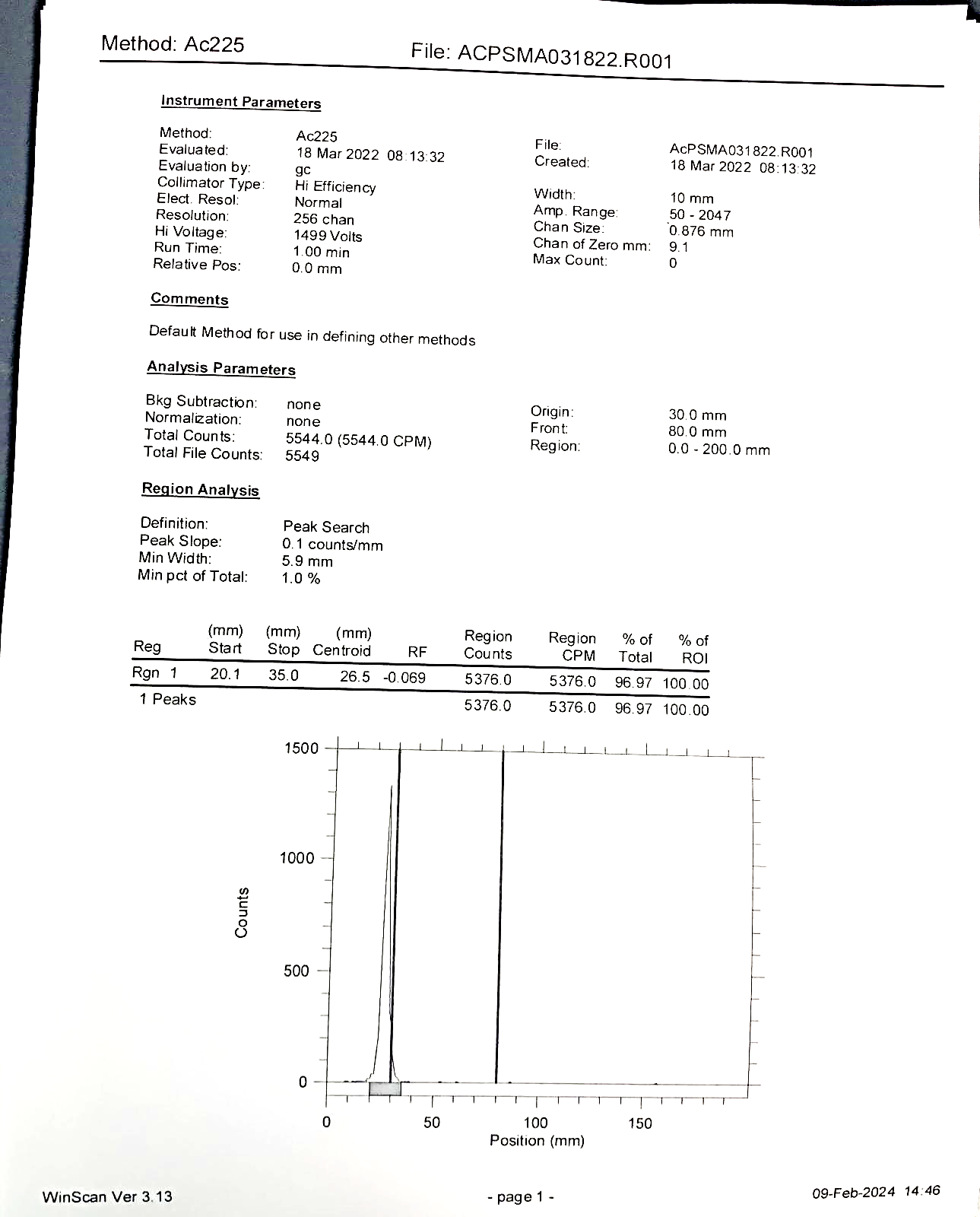


Position (mm)

**Fig S1:** ITLC of [^225^Ac]Ac-PSMA-617

Position (mm)

Counts


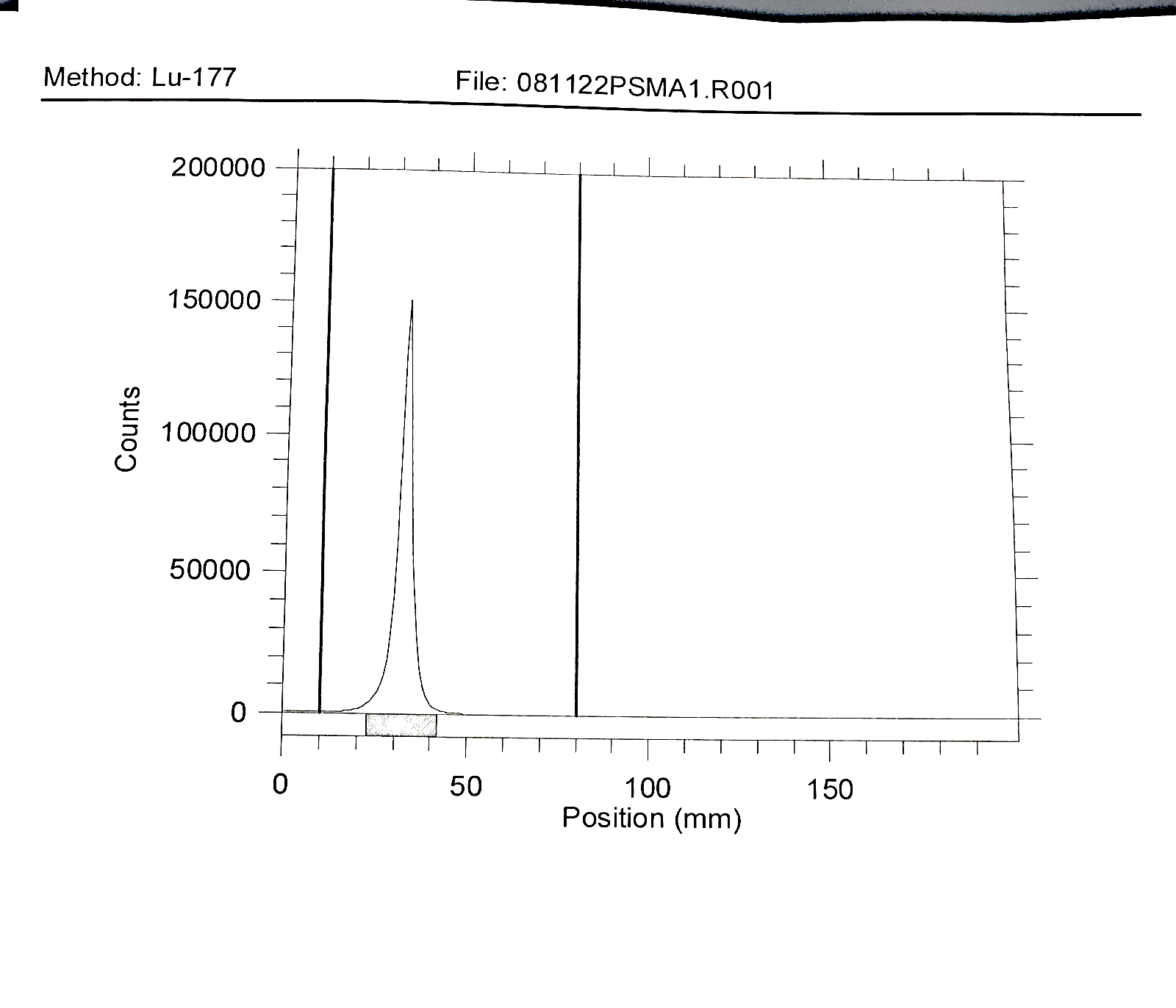


**Fig S2:** ITLC of [^177^Lu]Lu-PSMA-617


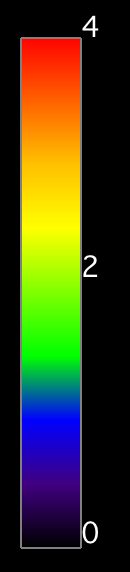

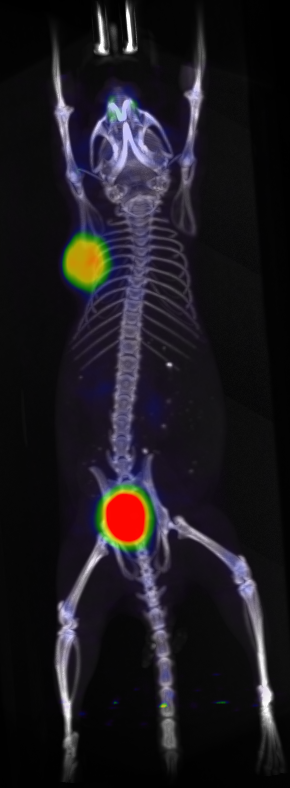


**Tumor: SUVmax = 2.76 ± 0.44**

**Fig S3**: Representative PET/CT scan [^68^Ga]Ga-PSMA-617; 1.1 MBq of control group RM1-PGLS tumor-bearing mouse before treatment. Tumor is indicated with a white arrow.

**Supplementary Table 1: Blood Cell Counts and Plasma Parameters**

| **Parameter** | **Control (Tumor)** | **[^225^Ac]Ac-PSMA-617 (Tumor)** | **[^225^Ac]Ac-PSMA-617 (No Tumor)** | **[^177^Lu]Lu-PSMA-617 (Tumor)** | **[^177^Lu]Lu-PSMA-617 (No Tumor)** |
| --- | --- | --- | --- | --- | --- |
| **White Blood Cell (x10^9^/L)** | | | | | |
| Day 4 | 5.3 ± 0.63 | 5.84 ± 0.55 | 5.2 ± 0.38 | 5.36 ± 0.50 | 5.25 ± 0.75 |
| Day 10 | 6.42 ± 1.07 | 5.36 ± 0.41 | 4.18 ± 0.24 | 2.5 ± 0.21 | 2.86 ± 0.30 |
| Day 15 | 6.9 ± 0.73 | 4.04 ± 0.05 | 3.4 ± 0.45 | 1.72 ± 0.26 | 1.94 ± 0.07 |
| Day 21 | 7.4 ± 1.4 | 4.02 ± 0.25 | 3.3 ± 0.30 | 1.87 ± 0.03 | 2.01 ± 0.06 |
| Day 28 | 7.63 ± 1.68 | 4.94 ± 0.82 | 5.04 ± 0.41 | 5.92 ± 0.92 | 2.04 ± 0.09 |
| Day 35 | 11.84 | 6.86 ± 0.42 | 4.36 ± 0.49 | 4.57 ± 1.38 | 2.18 ± 0.27 |
| Day 42 | 10.57 | 6.55 ± 0.65 | 5.72 ± 0.37 | 4.97 ± 1.86 | 2.0 ± 0.08 |
| Day 49 | - | 9.38 ± 1.67 | 5.38 ± 0.20 | 7.35 ± 0.95 | 3.74 ± 0.19 |
| Day 56 | - | 11.33 ± 3.18 | 5.9 ± 0.09 | 8.3 | 3.94 ± 0.38 |
| Day 63 | - | 10.33 ± 3.84 | 5.84 ± 0.07 | - | 5.88 ± 0.33 |
| **Lymphocytes (x10^9^/L)** | | | | | |
| Day 4 | 4.9 ± 0.60 | 5.22 ± 0.46 | 4.86 ± 0.33 | 4.98 ± 0.35 | 4.96 ± 0.75 |
| Day 10 | 5.14 ± 1.05 | 4.66 ± 0.59 | 3.78 ± 0.20 | 2.11 ± 0.22 | 2.74 ± 0.37 |
| Day 15 | 3.91 ± 0.56 | 3.58 ± 0.26 | 3.33 ± 0.45 | 1.2 ± 0.16 | 1.32 ± 0.17 |
| Day 21 | 3.23 ± 0.50 | 3.60 ± 0.44 | 3.20 ± 0.31 | 1.21 ± 0.13 | 1.76 ± 0.04 |
| Day 28 | 3.97 ± 0.44 | 4.14 ± 0.49 | 4.80 ± 0.45 | 1.34 ± 0.18 | 1.52 ± 0.18 |
| Day 35 | 2.21 | 4.02 ± 0.49 | 4.36 ± 0.66 | 1.8 ± 0.32 | 1.94 ± 0.33 |
| Day 42 | 3.96 | 4.46 ± 0.39 | 5.78 ± 0.40 | 1.04 ± 0.19 | 1.97 ± 0.16 |
| Day 49 | - | 4.86 ± 0.50 | 5.72 ± 0.29 | 3.15 ± 1.05 | 3.68 ± 0.18 |
| Day 56 | - | 9.45 ± 2.29 | 6.14 ± 0.25 | 2.11 | 3.72 ± 0.38 |
| Day 63 | - | 7.25 ± 5.25 | 6.88 ± 0.16 | - | 5.08 ± 0.36 |
| **Red Blood Cells (x10^12^/L)** | | | | | |
| Day 4 | 6.38 ± 0.41 | 6.52 ± 0.45 | 6.70 ± 0.18 | 6.72 ± 0.33 | 6.86 ± 0.44 |
| Day 10 | 6.84 ± 0.51 | 5.74 ± 0.42 | 6.12 ± 0.31 | 5.62 ± 0.18 | 6.32 ± 0.18 |
| Day 15 | 6.16 ± 0.22 | 6.49 ± 0.38 | 6.09 ± 0.40 | 5.30 ± 0.12 | 6.71 ± 0.26 |
| Day 21 | 6.38 ± 0.49 | 6.15 ± 0.43 | 5.81 ± 0.07 | 5.22 ± 0.21 | 5.16 ± 0.07 |
| Day 28 | 6.15 ± 0.16 | 5.72 ± 0.14 | 5.56 ± 0.06 | 5.28 ± 0.32 | 5.84 ± 0.09 |
| Day 35 | 6.34 | 5.96 ± 0.24 | 5.82 ± 0.04 | 5.37 ± 0.23 | 5.79 ± 0.08 |
| Day 42 | 5.96 | 5.91 ± 0.04 | 6.04 ± 0.49 | 5.29 ± 0.37 | 5.34 ± 0.56 |
| Day 49 | - | 5.47 ± 0.37 | 5.8 ± 0.31 | 5.28 ± 0.07 | 5.87 ± 0.17 |
| Day 56 | - | 5.94 ± 0.40 | 6.1 ± 0.41 | 5.32 | 5.82 ± 0.08 |
| Day 63 | - | 5.24 ± 0.23 | 5.56 ± 0.09 | - | 6.12 ± 0.14 |
| **Haemoglobin (g/dL)** | | | | | |
| Day 4 | 7.72 ± 0.69 | 8.36 ± 0.55 | 8.30 ± 0.18 | 8.14 ± 0.38 | 9.95 ± 0.96 |
| Day 10 | 8.59 ± 0.72 | 6.93 ± 0.59 | 7.92 ± 0.48 | 6.96 ± 0.31 | 7.83 ± 0.29 |
| Day 15 | 7.78 ± 0.38 | 8.37 ± 0.41 | 7.89 ± 0.54 | 7.18 ± 0.22 | 7.69 ± 0.36 |
| Day 21 | 7.81 ± 0.32 | 7.74 ± 0.48 | 7.32 ± 0.26 | 7.16 ± 0.34 | 7.22 ± 0.09 |
| Day 28 | 7.24 ± 0.26 | 7.26 ± 0.24 | 7.11 ± 0.09 | 7.76 ± 0.48 | 7.36 ± 0.08 |
| Day 35 | 7.48 | 7.48 ± 0.37 | 7.21 ± 0.12 | 7.23 ± 0.27 | 7.39 ± 0.1 |
| Day 42 | 7.42 | 7.31 ± 0.04 | 7.93 ± 0.74 | 7.14 ± 0.57 | 7.18 ± 0.99 |
| Day 49 | - | 7.19 ± 0.39 | 7.18 ± 0.11 | 7.19 | 7.37 ± 0.11 |
| Day 56 | - | 7.45 ± 0.41 | 7.92 ± 0.69 | 7.06 | 7.41 ± 0.07 |
| Day 63 | - | 7.14 ± 0.16 | 7.76 ± 0.10 | - | 8.19 ± 0.27 |
| **Platelets (10^9^/L)** | | | | | |
| Day 4 | 184 ± 22.9 | 196 ± 10.3 | 237 ± 37.4 | 218 ± 41.3 | 205.6 ± 18.2 |
| Day 10 | 223.4 ± 19.2 | 192 ± 13.7 | 223 ± 36.8 | 202.2 ± 2.4 | 212.6 ± 7.4 |
| Day 15 | 191.6 ± 7.28 | 173 ± 16.6 | 199 ± 42.9 | 91.4 ± 4.9 | 131.8 ± 12.3 |
| Day 21 | 258.4 ± 30.4 | 147.2 ± 9.34 | 106.8 ± 5.31 | 162.2 ± 13.3 | 108.8 ± 21.2 |
| Day 28 | 220.4 ± 13.9 | 119.5 ± 10.8 | 108 ± 29.6 | 225.6 ± 24.6 | 179.4 ± 19.8 |
| Day 35 | 258 | 189 ± 17.9 | 157 ± 3.85 | 208.3 ± 10.5 | 159.2 ± 11.3 |
| Day 42 | 274 | 178.6 ± 7.35 | 149.6 ± 36.4 | 188.7 ± 26.2 | 177.2 ± 13.3 |
| Day 49 | - | 205.6 ± 12.7 | 178.4 ± 51.9 | 195.5 ± 20.5 | 174.4 ± 9.3 |
| Day 56 | - | 246.3 ± 14.7 | 192.4 ± 24.6 | 292 | 167.4 ± 10.8 |
| Day 63 | - | 237.8 ± 5.91 | 146.7 ± 39.8 | - | 171.6 ± 14.2 |
| **Mean Platelet Volume (fL)** | | | | | |
| Day 4 | 5.84 ± 0.09 | 5.91 ± 0.09 | 5.81 ± 0.07 | 5.95 ± 0.07 | 5.96 ± 0.05 |
| Day 10 | 5.96 ± 0.08 | 5.99 ± 0.10 | 5.76 ± 0.06 | 6.16 ± 0.08 | 6.01 ± 0.11 |
| Day 15 | 5.86 ± 0.08 | 5.83 ± 0.13 | 6.06 ± 0.12 | 5.82 ± 0.08 | 5.93 ± 0.09 |
| Day 21 | 6.04 ± 0.18 | 6.07 ± 0.12 | 6.02 ± 0.16 | 7.21 ± 0.22 | 7.11 ± 0.09 |
| Day 28 | 6.01 ± 0.16 | 6.32 ± 0.23 | 6.21 ± 0.26 | 6.38 ± 0.23 | 6.35 ± 0.19 |
| Day 35 | 6.16 | 6.11 ± 0.14 | 6.08 ± 0.11 | 6.47 ± 0.25 | 6.14 ± 0.07 |
| Day 42 | 5.97 | 6.29 ± 0.18 | 6.14 ± 0.09 | 5.92 ± 0.11 | 6.18 ± 0.05 |
| Day 49 | - | 6.12 ± 0.11 | 6.09 ± 0.15 | 6.21 ± 0.06 | 6.15 ± 0.17 |
| Day 56 | - | 6.62 ± 0.16 | 6.13 ± 0.19 | 6.63 | 5.84 ± 0.04 |
| Day 63 | - | 6.46 ± 0.36 | 6.19 ± 0.23 | - | 6.09 ± 0.12 |
| **Platelet Distribution Width (fL)** | | | | | |
| Day 4 | 27.21 ± 0.58 | 27.17 ± 0.57 | 27.41 ± 0.24 | 27.43 ± 0.64 | 27.44 ± 0.64 |
| Day 10 | 26.29 ± 0.25 | 27.34 ± 0.27 | 26.72 ± 0.07 | 27.51 ± 0.22 | 26.97 ± 0.43 |
| Day 15 | 27.13 ± 0.39 | 26.22 ± 0.61 | 26.01 ± 0.37 | 25.81 ± 0.55 | 26.77 ± 0.25 |
| Day 21 | 28.33 ± 1.29 | 27.32 ± 0.37 | 26.69 ± 0.26 | 33.11 ± 0.56 | 33.96 ± 0.58 |
| Day 28 | 27.43 ± 1.39 | 28.41 ± 0.56 | 27.23 ± 0.30 | 29.61 ± 0.30 | 28.12 ± 0.37 |
| Day 35 | 28.14 | 26.73 ± 0.23 | 28.01 ± 0.69 | 27.79 ± 0.16 | 28.92 ± 0.49 |
| Day 42 | 27.23 | 28.32 ± 1.09 | 27.11 ± 0.28 | 28.3 ± 0.75 | 27.96 ± 0.26 |
| Day 49 | - | 27.35 ± 0.42 | 27.23 ± 0.33 | 27.56 ± 0.47 | 27.78 ± 0.47 |
| Day 56 | - | 28.73 ± 0.78 | 27.19 ± 0.29 | 28.6 | 26.78 ± 0.46 |
| Day 63 | - | 29.23 ± 1.27 | 28.07 ± 0.22 | - | 27.98 ± 0.27 |

**Supplementary Table 2: Blood Plasma Parameters**

| **Parameter** | **Control (Tumor)** | **[^225^Ac]Ac-PSMA-617 (Tumor)** | **[^225^Ac]Ac-PSMA-617 (No Tumor)** | **[^177^Lu]Lu-PSMA-617 (Tumor)** | **[^177^Lu]Lu-PSMA-617 (No Tumor)** |
| --- | --- | --- | --- | --- | --- |
| Creatinine (CRE) (µmol/L) | 42.5 ± 18.45 | 32.2 ± 8.18 | 24.4 ± 4.55 | 19 ± 0.45 | 19.46 ± 0.64 |
| Blood Urea Nitrogen (BUN) (mmol/L) | 16.75 ± 8.42 | 7.38 ± 0.24 | 8.4 ± 0.93 | 9.6 ± 0.42 | 9.2 ± 0.27 |
| Total Bilirubin (TBIL) (µmol/L) | 3.82 ± 0.01 | 4.23 ± 0.23 | 4.6 ± 0.40 | 4.5 ± 0.29 | 3.6 ± 0.51 |
| Alanine Transaminase (ALT) (U/L) | 75.5 ± 26.13 | 69.6 ± 30.36 | 72.8 ± 13.76 | 25 ± 0.41 | 88.4 ± 23.53 |
| Alkaline Phosphatase (ALP) (U/L) | 26.75 ± 5.44 | 39 ± 7.24 | 73.6 ± 3.31 | 26.4 ± 1.21 | 55.6 ± 4.86 |
| Aspartate Aminotransferase (AST) (U/L) | 575 ± 130 | 418 ± 81.45 | 119.4 ± 17.94 | 268.8 ± 27.7 | 174.8 ± 30.42 |
| Globulin (GLOB) (g/L) | 24 ± 3.51 | 20.6 ± 2.32 | 13.2 ± 0.71 | 24.75 ± 1.37 | 12.6 ± 0.81 |
| Albumin (ALB) (g/L) | 31 ± 3.79 | 27.8 ± 2.22 | 36.6 ± 0.95 | 25.25 ± 0.48 | 34.26 ± 0.34 |
| Total Protein (TP) (g/L) | 58.25 ± 3.35 | 48.4 ± 2.11 | 47.6 ± 0.51 | 49.5 ± 1.85 | 50.3 ± 0.42 |
